# Navigating chemical space: multi-level Bayesian optimization with hierarchical coarse-graining

**DOI:** 10.1039/d5sc03855c

**Published:** 2025-07-30

**Authors:** Luis J. Walter, Tristan Bereau

**Affiliations:** a Institute for Theoretical Physics, Heidelberg University Philosophenweg 19 69120 Heidelberg Germany bereau@uni-heidelberg.de; b Interdisciplinary Center for Scientific Computing (IWR), Heidelberg University Im Neuenheimer Feld 205 69120 Heidelberg Germany

## Abstract

Molecular discovery within the vast chemical space remains a significant challenge due to the immense number of possible molecules and limited scalability of conventional screening methods. To approach chemical space exploration more effectively, we have developed an active learning-based method that uses transferable coarse-grained models to compress chemical space into varying levels of resolution. By using multiple representations of chemical space with different coarse-graining resolutions, we balance combinatorial complexity and chemical detail. To identify target compounds, we first transform the discrete molecular spaces into smooth latent representations. We then perform Bayesian optimization within these latent spaces, using molecular dynamics simulations to calculate target free energies of the coarse-grained compounds. This multi-level approach effectively balances exploration and exploitation at lower and higher resolutions, respectively. We demonstrate the effectiveness of our method by optimizing molecules to enhance phase separation in phospholipid bilayers. Our funnel-like strategy not only suggests optimal compounds but also provides insight into relevant neighborhoods in chemical space. We show how this neighborhood information from lower resolutions can guide the optimization at higher resolutions, thereby providing an efficient way to navigate large chemical spaces for free energy-based molecular optimization.

## Abstract 2

Multi-level Bayesian optimization with hierarchical coarse-grained models enables efficient navigation of chemical space for molecular discovery, exemplified by the design of small molecules that enhance phase separation in phospholipid bilayers.

## Graphical abstract

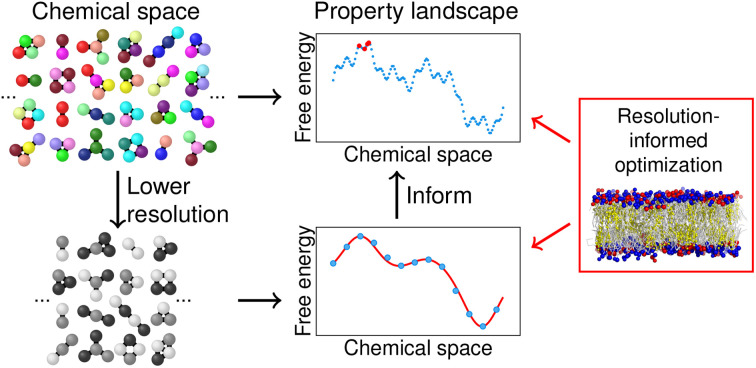

## 1 Introduction

All molecules consist of a limited set of atoms, but their diverse properties arise from the intricate arrangements of these atoms. The vast combinatorial possibilities of such arrangements define the so-called chemical space (CS).^1^ Exploring this space to discover new molecules with desired properties is challenging due to its immense size and complexity.^2,3^ Traditionally, experimental high-throughput screening is conducted on a small subset of molecular structures to identify candidates with the desired properties. However, this approach is costly and limited by the size of the molecular library.^4,5^

To address these challenges, computational methods have been employed to replace expensive experiments.^6^ In particular, molecular dynamics (MD) simulations can be utilized to predict the behavior of molecules based on their structure and empirical force fields.^7–9^ Combined with automated, high-throughput setups, they enable the screening of large numbers of molecules.^10^ While such simulations can reduce the cost of evaluating molecules for their target properties, they do not inherently facilitate navigation of the vast chemical search space.

Active learning methods—particularly Bayesian optimization (BO)—offer an efficient way to identify promising molecules from the extensive candidate pool. These methods optimize functions where gradient-based approaches are inapplicable.^11,12^ As molecular structure–property relationships generally lack gradient information, BO offers a more efficient alternative to uniform or random sampling of molecular space.^13–15^ Since BO relies on a covariance function over the input space, a numerical representation of discrete CS is typically used to quantify molecular similarity. For example, autoencoder models can encode molecules into latent representations.^16–18^ In contrast to fingerprint methods,^19–22^ they do not require a manual feature selection. Although BO helps select promising candidates, it does not reduce the complexity of CS.

Coarse-graining—grouping atoms into pseudo-particles or beads—addresses this complexity by effectively compressing CS. While traditionally employed to accelerate MD simulations, mapping atoms to beads reduces information and results in many-to-one relationships between atomistic and coarse-grained (CG) structures.^9,23^ The collective properties of the underlying chemical fragments determine the interactions between the CG beads. Discretizing these interactions enables the use of a transferable CG force field, *i.e.*, a fixed set of interaction or bead types that can be reused across the entire CS.^24^ The interaction resolution of such transferable force fields, determined by the number of available CG bead types, directly impacts the many-to-one relationship between atomistic and CG structures and therefore the combinatorial complexity of CG CS.^25^ Lower-resolution CS representations with fewer available bead types are easier to explore, but the resulting molecular structures lack detailed information.^26^ Higher resolutions provide more detailed results, but their CS representations are more challenging to explore. This raises the question of how to combine different coarse-graining resolutions to efficiently explore CS while obtaining detailed molecular results.

In this work, we propose a multi-level BO framework for an efficient exploration of small molecule CS across multiple CG force-field resolutions. Our method combines the reduced complexity of CS exploration at lower resolutions with a detailed optimization at higher resolutions. The Bayesian approach provides an intuitive way to combine information from different resolutions into the optimization. Our method builds upon the work of Mohr *et al.*, who applied BO in a single, relatively low-resolution CG representation of CS to derive molecular design rules.^26^ They also conducted optimization in a learned representation of an enumerated CG CS. We build on their approach by integrating multiple CG resolutions into a unified optimization framework.

Our multi-level BO is related to previous multi-fidelity BO efforts,^27–30^ which rely on different evaluation costs and accuracies for each fidelity. In contrast, we assume a constant evaluation cost at all levels and instead utilize the varying complexity of our different CG resolutions.

Compared to recently popular generative methods for inverse molecular design,^31^ our multi-level BO framework is data efficient and requires no prior training data for the optimization target.

As a demonstration of our method, we optimize a small molecule to promote phase separation in a ternary lipid bilayer. Previous studies^32,33^ have shown that molecules embedded within lipid bilayers can modulate their phase behavior. We quantify this phase separation behavior as a free-energy difference, which serves as the objective function for our molecular optimization. We demonstrate that our multi-level BO algorithm effectively identifies relevant chemical neighborhoods and outperforms standard BO applied at a single resolution level. Our proposed approach is versatile and applicable to a broad range of small-molecule optimization tasks where the target property can be expressed as a free-energy difference.

## 2 Methods

### 2.1 Overview

**Fig. 1:**
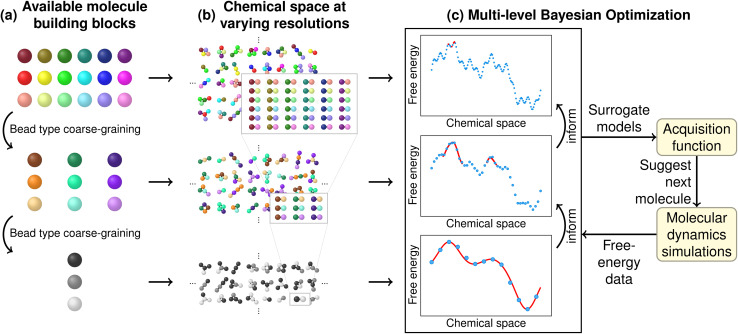
Overview of our multi-resolution coarse-graining molecule optimization workflow. (a) Definition of multiple coarse-grained (CG) models at varying resolutions. These models share the same atom-to-bead mapping but differ in bead-type assignments, with higher resolutions featuring more bead types to capture finer chemical details. (b) Enumeration of chemical space (CS) at different resolution levels. Higher-resolution molecules can be hierarchically mapped to lower resolutions. (c) Multi-level Bayesian optimization integrating information from all CS resolutions. Molecules are iteratively suggested by an acquisition function and evaluated through molecular dynamics (MD) simulations. The optimization progressively shifts toward higher-resolution evaluations. Optimization at higher-resolution levels is guided by surrogate models at lower resolutions, improving efficiency and accelerating the search for optimal candidates.

We begin by providing an overview of our computational screening methodology. First, we defined multiple CG models with varying resolutions, all using the same atom-to-bead mapping but differing in the assignment of transferable bead types. Higher-resolution models featured more bead types, capturing finer chemical details while still reducing the combinatorial complexity of CS compared to the atomistic level (Fig. 1a). This reduction allowed us to enumerate all possible CG molecules corresponding to a specific region of CS at each resolution. Due to the hierarchical model design, higher-resolution molecules could be systematically mapped to lower resolutions (Fig. 1b).

For the next step of our molecule optimization, we embedded the CG structures into a continuous latent space using a graph neural network (GNN)-based autoencoder, with each resolution encoded separately. This encoding step provided a smooth representation of CS, ensuring a meaningful similarity measure necessary for the subsequent BO.

Finally, a multi-level Bayesian optimization was performed based on all previously encoded CS resolutions. The ground truth values, *i.e.*, the optimization targets, were obtained from MD simulation-based free-energy calculations (Fig. 1c). In our example application, such a free-energy estimate characterized the phase separation behavior of a molecule inserted into a ternary lipid bilayer. The following sections describe each of the molecular discovery steps in detail.

### 2.2 Multi-resolution coarse-graining of CS

Coarse-graining of molecules generally consists of two steps. First, groups of atoms are mapped to pseudo-particles or beads. Second, the interactions between these beads are defined based on their underlying atomistic fragments. For both steps, the resolution of the coarse-graining can be varied. Assigning larger groups of atoms to single beads results in a lower CG resolution for the mapping step. Interactions between beads can be defined for each bead pair^34,35^ or discretized into a limited number of transferable bead types. The number of available bead types then defines the interaction resolution. Various CG models with different approaches to the mapping, discretization, and assignment of bead types exist.^36,37^

Since coarse-graining reduces information, a single CG molecule corresponds to multiple atomistic conformations or chemical compositions. The CG resolution determines how many atomistic structures correspond to a single CG molecule. Representing CS at a lower CG resolution results in fewer combinatorial possibilities for molecules and therefore a smaller CS.^25^

We started the molecule discovery process by directly defining small molecule CS at the high-resolution CG level. To do this, we specified the set of available CG bead types based on the relevant elements and chemical fragments from atomistic CS (Fig. 1a). We used three CG resolution levels for our application. They shared the same mapping of atoms to beads, but differed in the number of available bead types. Our high-resolution model corresponded to the Martini3 model,^24^ a versatile CG force field with demonstrated relevance to materials design.^26,38,39^ For our model, we ignored Martini3 bead labels, *e.g.*, for hydrogen bonding or polarizability. Further excluding water and divalent ions resulted in a model with 32 bead types per bead size, or 96 bead types in total. The relationship between bead types at different resolutions was hierarchical, meaning that higher-resolution bead types could be uniquely mapped to lower resolutions. In practice, lower-resolution bead types were obtained by averaging the interactions of higher-resolution bead types. For the medium- and low-resolution models, we derived 45 and 15 bead types, respectively. Section S1.1 of the SI provides further details on the derivation of lower-resolution models.

For all resolutions, we enumerated all possible CG molecules based on the available bead types and the defined molecule size limit of up to four CG beads (Fig. 1b). By directly generating molecules at the CG level, the atomistic resolution was bypassed. Since we assumed bead size-dependent but constant bond lengths and no angle or dihedral interactions, the enumeration of molecules is equivalent to the enumeration of graphs. The small molecule size justified the neglected angle and dihedral interactions. For the three levels of resolution, we obtained chemical spaces of approximately 90 000, 6.7 million, and 137 million molecules, respectively. Section S1.2 of the SI elaborates details on the graph enumeration.

### 2.3 Chemical space encoding

From the enumeration step, we obtained large sets of molecular graphs. While direct optimization in graph space is possible (*e.g.*, *via* evolutionary algorithms^40–42^), a numerical representation facilitates exploration of CS by enabling distance-based similarity measures. Molecular fingerprints are often used for this purpose^19–22^ but require manual feature selection. Instead, we used a learned projection of CS into a low-dimensional, smooth numerical representation.

For the learned encoding, we used a regularized autoencoder (RAE),^43^ which offers deterministic behavior compared to the more common variational autoencoder (VAE) architecture.^16,44^ As we only aimed for a smooth embedding, the stochasticity of a VAE was not needed. The built-in regularization of the RAE ensured a well-structured latent space.^43^ We used a GNN for the node-permutation invariant encoder,^45,46^ which mapped molecular graphs to the five-dimensional latent space. A decoder, composed of fully connected layers, was used to reconstruct node features and the adjacency matrix. Although the decoder was not invariant to node permutations, the reconstruction loss ensured an invariant training of the RAE.

Input and reconstruction node features included bead-type class, size, charge, and octanol–water partition coefficient. The latter was added as a continuous feature to improve latent space structure.

We trained separate RAEs for each CG resolution using the complete set of enumerated molecules. The separated training resulted in lower reconstruction losses and better adaptation to the reduced resolution at lower levels. The loss combined cross-entropy terms for categorical features, a binary cross-entropy for the adjacency matrix, and a mean squared error term for the octanol–water partition coefficient. After training, we retained only the encoder for embedding molecules. The RAE was implemented using the PyTorch and PyTorch Geometric libraries,^47,48^ following the architecture of Mohr *et al.*^26^ Further details on the RAE architecture, the training, and an analysis of the learned latent space are provided in Section S1.3 and S2.1 of the SI. In the following steps, we performed BO in these learned latent spaces.

### 2.4 Single-level Bayesian optimization

Before introducing our multi-level BO approach, we first provide an overview of standard BO and our notation (see, *e.g.*, Frazier^12^ for a more detailed introduction). We then describe how we extend this approach to combine multiple resolution levels into a single optimization process. BO aims to optimize a black-box function 
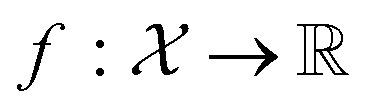
 that is expensive to evaluate and has no analytical form or gradient information available. The objective is to find the global optimum 
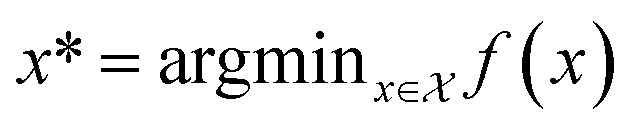
 or 
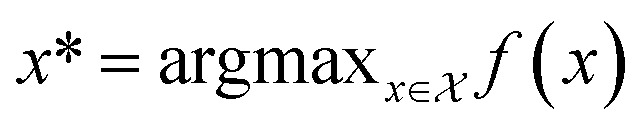
 with as few function evaluations as possible. Typically, a Gaussian process (GP) is used as a probabilistic model for *f*(*x*), *i.e.*, 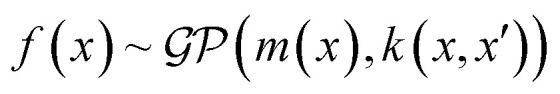, defining a multivariate normal distribution with mean function *m*(*x*) and a covariance function *k*(*x*, *x*′). This covariance kernel quantifies correlations over 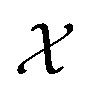. Although various kernel functions exist, a common choice is the radial basis function (RBF) kernel, defined as1
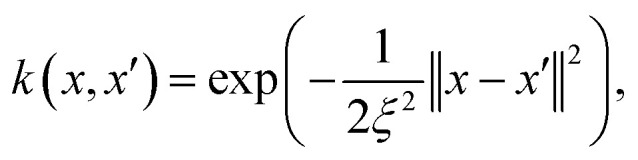
where *ξ* is the length scale parameter. Given training data 
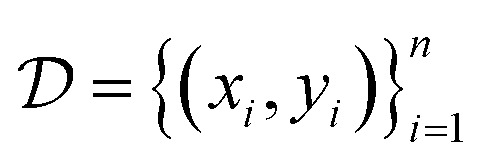
 with inputs *X* = {*x*_1_,…, *x*_*n*_} and observations *Y* = {*y*_1_,…, *y*_*n*_}, the posterior GP provides a predictive mean *μ*(*x*) and variance *σ*^2^(*x*) for any 
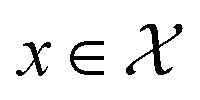
. The mean and variance are given by

2 *μ*(*x*) = *m*(*x*) + *k*(*x*, *X*)*K*^−1^(*Y* − *m*(*X*)),

3 *σ*^2^(*x*) = *k*(*x*, *x*) − *k*(*x*, *X*)*K*^−1^*k*(*X*, *x*),

where 
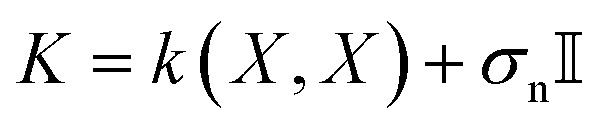
 is the covariance matrix of *X* with an added noise term *σ*_n_.

In BO, the GP model is iteratively updated with new evaluations of the target function. First, the function is evaluated at a set of initialization points. Subsequent evaluations are selected based on the predictive mean and variance of the GP, guided by an acquisition function that balances exploration and exploitation. A common choice for the acquisition function is the expected improvement (EI),^49^ which for minimization is defined as4
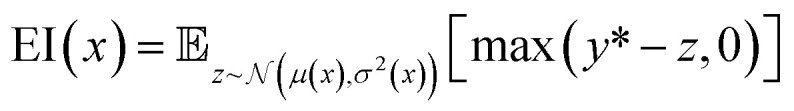
with y* = min_*y*∈*Y*_*y*. The next evaluation point is determined by 
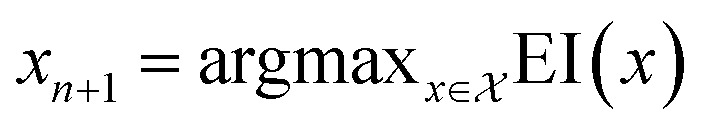
. This process repeats until the evaluation budget is exhausted or a sufficiently good solution is found.

### 2.5 Multi-level Bayesian optimization

For our multi-level BO approach, we considered *d* = 3 CG resolution levels of CS. At each level *l* ∈ {1,…, *d*}, we defined the mapping of chemical space 
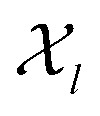
 to the target free-energy difference *y* as an unknown function *f*_*l*_(*x*). Our goal was to identify molecules at the highest resolution *d* that are near the optimum, *i.e.*, 
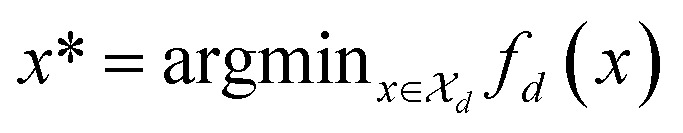
, while leveraging information from the lower-resolution models (*l* < *d*). Similar to the work of Huang *et al.*, we assumed that each function *f*_*l*_(*x*) can be modeled as a correction to the lower resolution

5 *f*_*l*_(*x*) = *f*_*l*−1_(*x*) + *δ*_*l*_(*x*),

where *δ*_*l*_(*x*) represents the correction term.^27^ The hierarchical bead-type resolutions justified this delta learning approach. We modeled each *δ*_*l*_(*x*) as a GP, *i.e.*,6
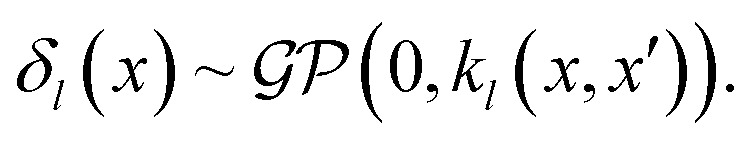
with a mean function equal to zero for all *x*. For all levels, we used an RBF kernel function (eqn (1)) with level-specific length scale parameters *ξ*_*l*_. By definition of the GP (see eqn (2) and (3)), this delta learning approach corresponds to a GP with a mean prior *m*(*x*) equal to the next-lower resolution function *f*_*l*−1_(*x*). Thus, we can rewrite the GP for *f*_*l*_(*x*) as7
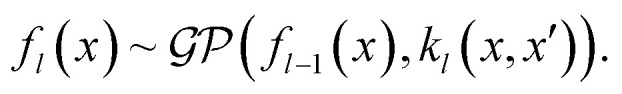
At the lowest resolution *l* = 1, no lower-level prior was available. Instead of using a zero prior for *f*_1_(*x*), we applied a simple model *f*_0_(*x*) that approximates the free-energy difference of a molecule as the sum of the individual bead free energies.

Until now, we assumed the latent spaces of the different resolutions to be compatible. However, since they were obtained from separate autoencoder trainings, we could not directly use a lower level function *f*_*l*_(*x*) as the prior for the GP on level *l*. Instead, a function 
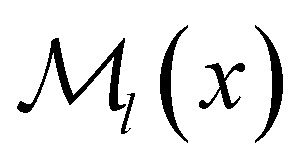
 was required that maps points in latent space 
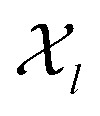
 to points in latent space 
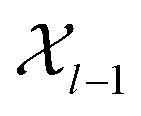
. We determined this mapping from one resolution to a lower one from the known relationships between molecules at different resolutions. Effectively, we had a many-to-one mapping from 
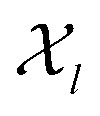
 to 
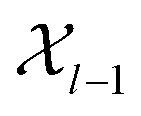
, which made the mapping 
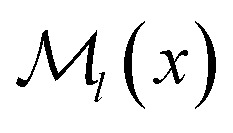
 an unambiguous function. Applying this mapping to eqn (7), we get8
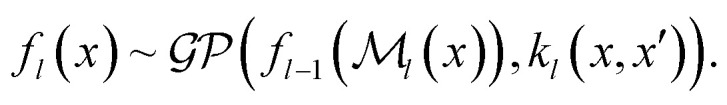
as the final probabilistic model for resolution *l*.

The optimization procedure started at the lowest-resolution level *l* = 1, with initialization molecules selected through weighted *k*-medoid clustering of the encoded CS. The clustering weights were based on the prior of the lowest resolution and calculated as *w*_*i*_ = exp(−*f*_0_(*x*_*i*_)).

The length scale parameters *ξ*_*l*_ of the RBF kernels were optimized for each level using the GP marginal likelihood. The kernel noise term *σ*_n_ from *K* in eqn (2) and (3) was fixed to the standard deviation of the calculated free-energy differences. This standard deviation was determined by multiple repeated evaluations of the same molecule (see Section S2.3 of the SI). The multi-level BO implementation used the GPyTorch library.^50^

Although BO is also possible with a batched evaluation of multiple points,^26^ we only evaluated one point, *i.e.*, one molecule, at a time. Since each evaluation involved multiple MD simulations, we parallelized over these simulations. We used the EI as the acquisition function on each level. For higher levels *l* > 1, the EI was computed and maximized only over CS regions with expected significant negative free-energy differences. These regions were defined as the neighborhoods of points with promising prior information from the lower level. Restricting the EI calculation to these neighborhoods focused the optimization on the most relevant CS regions and accelerated the EI maximization process. Details regarding the mapping of points between latent spaces and the calculation of neighborhoods are provided in Section S1.4 of the SI.

Our multi-level BO algorithm transitions to a higher resolution when the prediction error of the GP remains below a predefined threshold for multiple consecutive evaluations. This prediction error serves as a measure of the GP model's convergence. For our application, we empirically set the prediction error threshold to 0.12 kcal mol^−1^ and required three consecutive evaluations below this threshold to trigger the switch. These hyperparameters control the trade-off between exploration at lower resolutions and faster exploitation of promising regions at higher resolutions. Lowering the threshold and increasing the number of required evaluations enhances exploration at lower resolutions, but increases the total number of molecule evaluations needed.

In addition to increasing the resolution level, the algorithm can switch back to the previous lower resolution. Since we want to effectively leverage lower-resolution models, we are only interested in high-resolution evaluations in regions where a reliable prior is available. If the candidate with the maximal EI is too far away from regions with a reliable prior from lower levels, we switch back to the previous resolution level. Specifically, the criterion for switching to resolution level *l* – 1 is defined as ‖x* – *x*′‖ > 2*ξ*_*l*_, 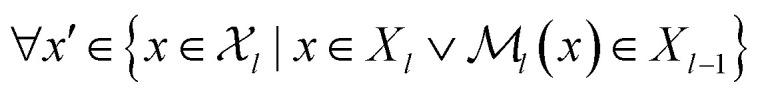, where *X*_*l*_ denotes the set of already evaluated points at level *l*.

### 2.6 Estimating the membrane demixing behavior

**Fig. 2:**
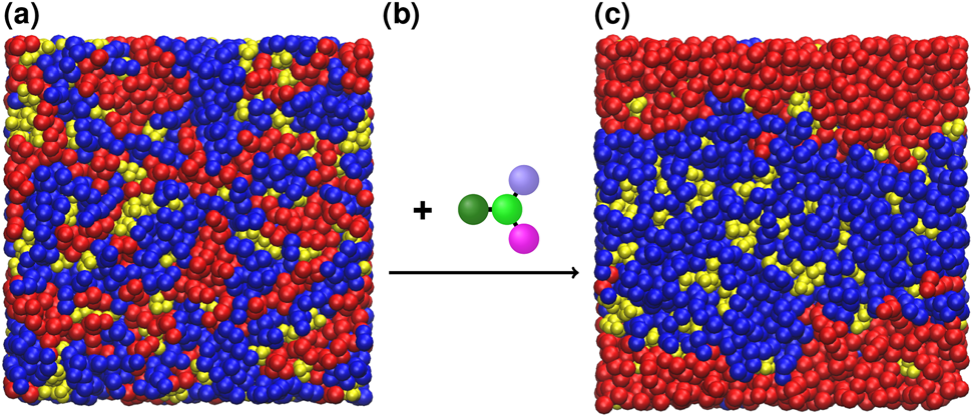
Influencing phase separation in a lipid bilayer by inserting small molecules. Shown is a top view of a CG ternary lipid bilayer composed of DPPC (blue), DLiPC (red), and cholesterol (yellow). (a) In the mixed state, the bilayer contains small, dispersed lipid patches. (b) Upon inserting specific small molecules, (c) the bilayer transitions to a demixed state with pronounced phase separation between the two phospholipids.

**Fig. 3:**
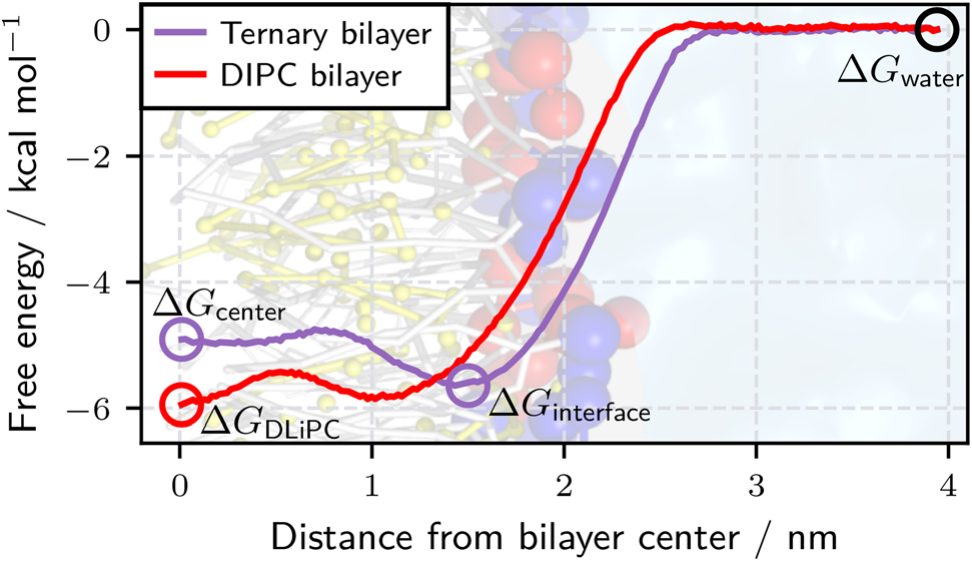
Estimating the demixing behavior of molecules *via* free-energy calculations at four bilayer depths (circles) as an alternative to potential of mean force (PMF) computations (solid lines). For the molecule optimization, we aim to minimize ΔΔ*G* = Δ*G*_center_ − Δ*G*_DLiPC_ under the conditions that Δ*G*_center_ < Δ*G*_interface_ and Δ*G*_center_ < Δ*G*_water_. The background illustrates the hydrophobic tails (grey), the charged headgroups of DPPC (blue) and DLiPC (red), as well as cholesterol (yellow). The plotted PMFs correspond to a molecule with Δ*G*_center_ > Δ*G*_interface_, indicating that it localizes at the bilayer interface and therefore does not significantly influence lipid mixing.

For our application, we optimized small molecules to enhance phase separation in a ternary lipid bilayer consisting of 1,2-dipalmitoyl-*sn*-glycero-3-phosphocholine (DPPC), 1,2-dilinoleoyl-*sn*-glycero-3-phosphocholine (DLiPC), and cholesterol (Fig. 2). DPPC and DLiPC differ only in their acyl chains, with DPPC having two saturated 16-carbon chains and DLiPC having two doubly unsaturated 18-carbon chains. The phase separation can be quantified by the DPPC–DLiPC contact fraction.^32^ However, directly observing the effect of a molecule on lipid mixing requires long simulations with large bilayer leaflets, which is impractical for high-throughput screening. Alternatively, potential of mean force (PMF) profiles along the axis perpendicular to the bilayer plane can be compared for pure DPPC, DLiPC, and ternary bilayers.^33^ Since PMF calculations (*e.g.*, *via* umbrella sampling^51^) are still computationally expensive, we employed thermodynamic integration (TI)^52,53^ calculations at a few key positions in the bilayers as a proxy. Centi *et al.*^33^ showed that molecules that influence the demixing or mixing of a DPPC–DLiPC bilayer localize near the bilayer center because the two phospholipids differ only in their carbon tails. To determine a molecule's preferred localization, we performed TI computations at the center (*z* = 0 nm) of the ternary bilayer, at the interface (*z* = 1.5 nm) and in bulk water (Fig. 3), obtaining the free-energies Δ*G*_center_, Δ*G*_interface_, and Δ*G*_water_, respectively. We initially used Δ*G*_center_ and Δ*G*_water_ to identify non-inserting molecules, allowing us to skip further free-energy evaluations for these cases. Centi *et al.*^33^ showed that molecules that enhance the phospholipid demixing localize near the DLiPC phase. Therefore, we performed a fourth TI calculation at the center of a pure DLiPC bilayer, yielding Δ*G*_DLiPC_. Unlike the direct observation of DPPC–DLiPC contacts, Δ*G*-based scoring was easily parallelized, thereby further reducing the wall time per evaluated molecule. The main optimization target was the free-energy difference, ΔΔ*G* = Δ*G*_center_ − Δ*G*_DLiPC_, assuming the molecule localizes near the ternary bilayer center. To ensure robust optimization even when molecules localize at the interface or in the water, we combined ΔΔ*G* with a score *S* defined as a conditional weighted sum of Δ*G*_water_ − Δ*G*_interface_ and Δ*G*_interface_ − Δ*G*_center_. In contrast to using a constant ΔΔ*G* for interface- or water-localizing molecules, this score provided a more nuanced direction for optimization, steering it toward relevant regions of CS. Negative ΔΔ*G* values indicated a preference for the DLiPC phase, corresponding to a demixing behavior. Overall, the molecule optimization corresponded to a minimization of min(ΔΔ*G*, 0) + *S*. Section S1.5 of the SI provides further information on the calculation of the score *S*.

### 2.7 Molecular dynamics simulations

We used MD simulations in a high-throughput manner^54,55^ to perform the TI calculations of the free-energy differences. All MD simulations were performed using GROMACS 2024.2.^56,57^ Martini3 and Martini3-derived (see Section 2.2) force fields were used for the CG simulations.^24,58^ The derived lower-resolution bead types are compatible with the standard Martini3 bead types and can therefore be evaluated within unmodified Martini3 environments.

Our lipid bilayer simulation setup was based on the protocol by Ozturk *et al.*^59^ We used a leap-frog stochastic dynamics integrator with an integration time step of 20 fs (in reduced CG units). All simulations were performed in the NPT ensemble at a temperature of 305 K and pressure of 1 bar,^33^ controlled by a semi-isotropic C-rescale barostat.^60^ For the TI, we used 26 linearly-spaced *λ* steps for the decoupling of Lennard-Jones interactions and 10 additional linear *λ* steps for the decoupling of Coulomb interactions in the case of charged molecules. Since each molecule evaluation required up to four TI calculations, each with up to 36 *λ* steps, evaluating a single molecule could require up to 144 individual simulations. Further simulation parameters are provided in Section S1.6 of the SI. The package MBAR^61,62^ was used to calculate free-energy differences from the MD simulation data.

Membrane systems were generated using the program insane.^63^ Following the approach of Centi *et al.*, we used a lipid composition of DPPC : DLiPC : cholesterol in a 7 : 4.7 : 5 ratio.^33^ For a bilayer area of 6 × 6 nm^2^, used for the free-energy evaluations, this corresponded to 26 DPPC, 18 DLiPC, and 19 cholesterol molecules per bilayer leaflet. We used the colvars module^64^ in GROMACS to calculate or restrain the phospholipid contact fraction. Specifically, the collective variable was defined as the coordination number between the first C1 beads of DLiPC and DPPC with a cutoff distance of 1.1 nm.^33^ During the TI simulations, the coordination number was restrained to 65 contacts per leaflet, yielding an average of 2.5 DLiPC molecules within the cutoff per DPPC. This slightly exceeds the 2.15 contacts expected from random lipid placement by insane.^63^

## 3 Results and discussion

### 3.1 Multi-level Bayesian optimization

We applied our multi-level BO workflow to identify small molecules that enhance the phase separation of a ternary lipid bilayer, demonstrating its effectiveness in navigating chemical space. We restricted the search to small molecules with up to 16 heavy atoms, corresponding to a maximum of four beads in our CG model. We imposed no additional constraints, such as the presence of specific functional groups, to rigorously test our method. Our multi-level molecule optimization utilized three coarse-graining resolutions, incorporating 15, 45, and 96 distinct bead types. While all three levels use the same spatial coarse-graining, complexity increased with the combinatorial diversity of bead types, spanning approximately 90 000, 6.7 million, and 137 million possible CG molecules. To identify phase separation-enhancing molecules at the highest resolution, we used lower-resolution models only to guide the search, thereby reducing the complexity of the optimization compared to direct high-resolution exploration. At all levels, a molecule's effect on phase separation was quantified by an MD simulation-derived free-energy difference, ΔΔ*G* (see Section 2.6).

The optimization was conducted within RAE-learned latent embedding spaces, generated from the CG models at each resolution. As a first step, we computed the ΔΔ*G* values for all 15 low-resolution bead types. These results enabled us to construct a cost-effective prior for the low-resolution model, based on an additivity assumption over individual bead values (see Section S2.1 of the SI for a detailed evaluation of this assumption). Using this prior, we initialized the multi-level active learning with 50 low-resolution molecules. Subsequent molecules and their resolution levels were determined iteratively by our multi-level BO algorithm. We evaluated 327 molecules in total: 106 molecules (15 + 50 + 41) at the low resolution, 148 at the medium resolution, and 73 at the high resolution. In each iteration, a single molecule was selected for evaluation using MD simulations. The resulting ΔΔ*G* value was then used to update the BO model, which informed the selection of the following molecule.

**Fig. 4:**
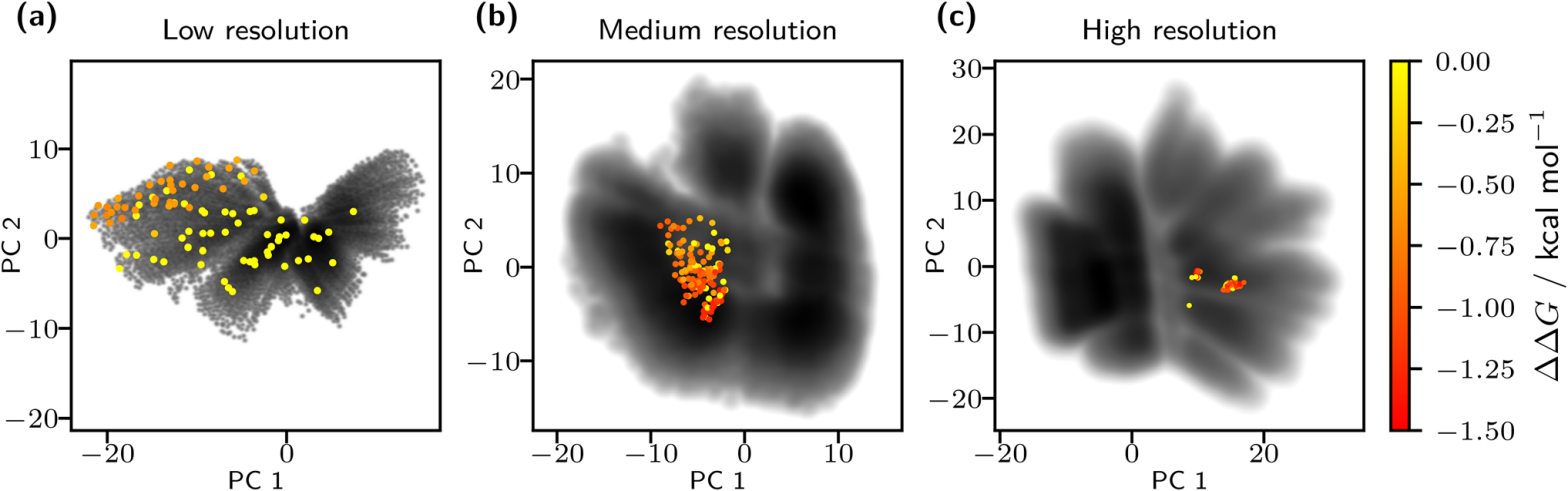
Encoded chemical spaces and evaluated points for the three levels of resolution. The full chemical spaces are shown as kernel-density estimations of latent space principal component analysis (PCA) projections (black). Evaluated molecules across the three resolutions are overlaid as colored points (yellow to red), where lower ΔΔ*G* values indicate stronger lipid bilayer demixing. Due to separate encodings at each resolution, latent space points are not directly transferable. (a) Optimization proceeds from broad, low-resolution exploration to (b) progressively focused searches in medium and (c) high resolutions.

**Fig. 5:**
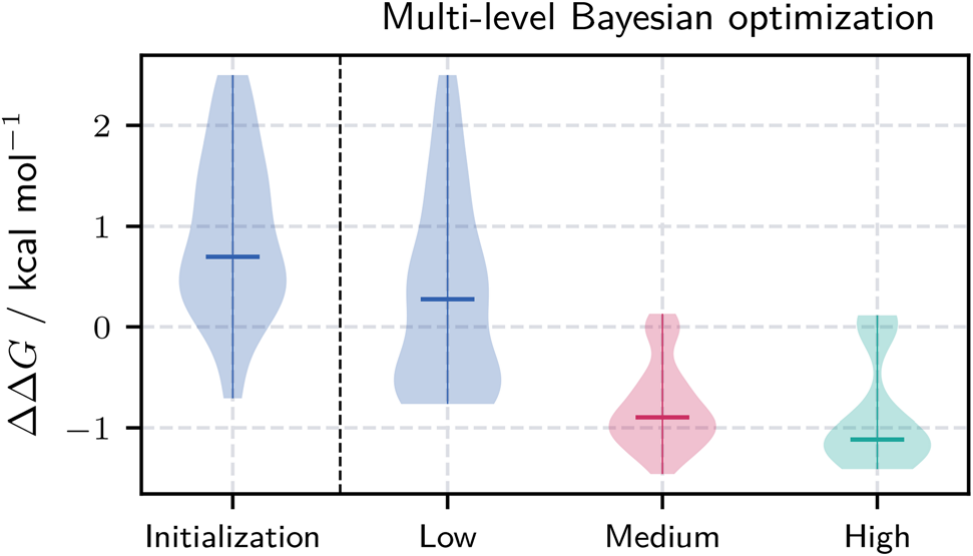
Distribution of ΔΔ*G* values for all evaluated candidates at different stages of the multi-level Bayesian optimization process. Violin plots show the distributions for the initialization set and candidates evaluated at low, medium, and high-resolution levels. As the optimization progresses to higher resolutions, the distribution of ΔΔ*G* values progressively shifts toward lower (more favorable) values. Horizontal bars indicate the median of each distribution.

Our multi-level BO approach progressively narrows the search space through the three resolution levels. The optimization begins with a broad exploration of low-resolution CS, identifying coarse regions likely to contain molecules with favorable ΔΔ*G* values. Insights from this stage inform the medium-resolution search, allowing the algorithm to focus on more promising sub-regions. This process is further refined at the high-resolution level to pinpoint localized areas within CS that are most likely to yield effective candidates. By leveraging information from the preceding levels, the algorithm bypasses large areas of the CS landscape that are unlikely to yield relevant molecules. Therefore, the number of required evaluations and the overall computational cost are reduced. Fig. 4 presents 2D projections of the encoded CS (black) together with the evaluated molecules. Because each resolution is encoded independently, the representations differ and prevent a direct transfer of points. However, molecules can be readily mapped across latent spaces by leveraging the known mapping between bead types. The figure illustrates the funnel-like optimization: as resolution increases, the search becomes more focused, eventually concentrating on localized sub-regions of chemical space. Many low-resolution candidates display unfavorable ΔΔ*G* values or negligible effects on phase separation (yellow). In contrast, searches at medium and high resolutions increasingly yield molecules with lower ΔΔ*G* values corresponding to a more substantial impact on lipid demixing (orange to red). Fig. 5 further illustrates this trend, showing the distribution of evaluated ΔΔ*G* values across the three resolution levels, including the initialization points at resolution *l* = 1. Candidates from the low-resolution optimization already show lower ΔΔ*G* values relative to the initialization set. However, higher-resolution candidates generally exhibited even stronger phase-separation effects, with medium resolution peaking around −1 kcal mol^−1^ and high resolution around −1.2 kcal mol^−1^. The differences between the low- and medium-resolution minima support our hypothesis about the varying smoothness of the free-energy landscape across resolutions.

The computational cost per simulation is the same across all three resolutions. Consequently, the overall computational load at each level is primarily determined by the number of evaluated molecules. For non-inserting molecules, two of the four TI calculations can be omitted (see Section 2.6). As the lowest resolution filtered out most non-inserting molecules, its average computational load per evaluation was slightly lower than at higher resolutions.

We terminated the optimization after 73 high-resolution evaluations, as no further improvement in ΔΔ*G* was observed. The 327 evaluated molecules correspond to less than 3 × 10^−4^% of the total high-resolution molecule space. While global optimality is not guaranteed, the workflow identified multiple promising candidates with pronounced effects on lipid phase separation despite limited evaluations.

### 3.2 Evaluation of optimized molecules

**Fig. 6:**
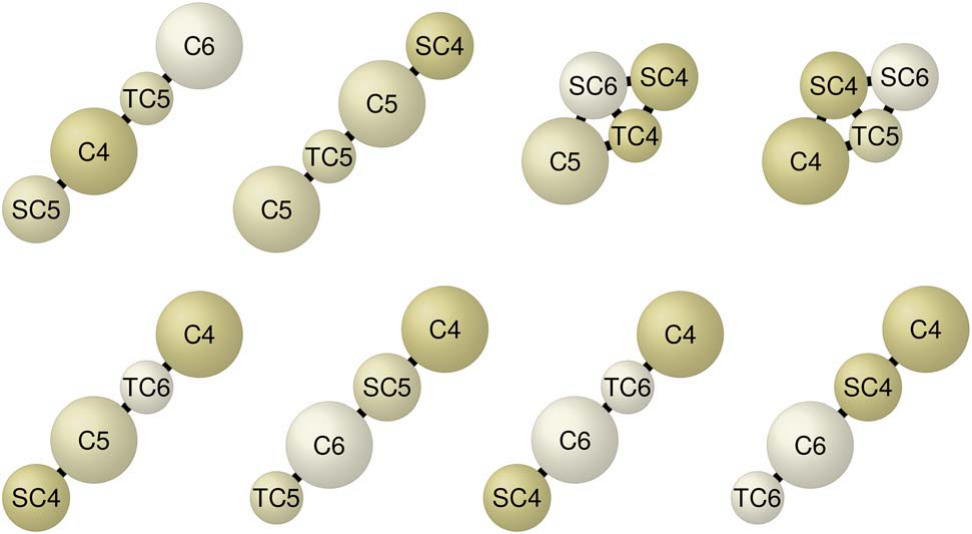
CG structures of the best eight high-resolution molecule candidates identified in the optimization process. The molecules exhibit low free-energy values (ΔΔ*G*) below −1.3 kcal mol^−1^, indicating a strong influence on phospholipid bilayer phase separation. All molecules are exclusively composed of hydrophobic C4, C5, and C6 beads (in different sizes, indicated by prefixes S/T), corresponding to Martini3 types for alkenes, aromatic rings, and sulfide groups, respectively. Six of the eight molecules have an extended/chain-like topology.

Following the overall optimization process analysis, we now focus on the top candidate molecules with the lowest ΔΔ*G* values. As the Martini3 CG model (without bead labels)^24^ corresponds to our high-resolution model, the optimized molecules do not provide atomistic details but reveal valuable insights into the chemical moieties driving the phospholipid phase separation. The top eight CG molecules, shown in Fig. 6, all display ΔΔ*G* values below −1.3 kcal mol^−1^, with the best candidate at −1.4 kcal mol^−1^ (top left of the figure). These results indicate a strong effect on the phase separation. A consistent feature across all eight CG molecules is the exclusive presence of hydrophobic C4, C5, and C6 beads in varying bead sizes. These Martini3 beads correspond to alkenes, aromatic rings, and thiol/sulfide groups, respectively.^24^ This observation aligns with Barnoud *et al.*, who showed that aromatic groups promote demixing, while aliphatic groups (C1, C2, and C3 beads) favor phospholipid mixing.^32^ The two distinct topologies shown in Fig. 6 correspond to the two prominent point clusters in the 2D projection of Fig. 4c. While each cluster contains molecules with a variety of topologies, the highest-scoring molecules within them are predominantly of the two topologies in Fig. 6.

The highest-performing molecules at both low and medium resolution (see Section S2.4 of the SI) exhibit more diverse topologies but share similar trends in bead-type composition. While the low-resolution results already provide preliminary chemical insights, more detailed information—such as the unfavorable contribution of C1, C2, and C3 beads—only becomes evident through the inclusion of higher-resolution models.

**Fig. 7:**
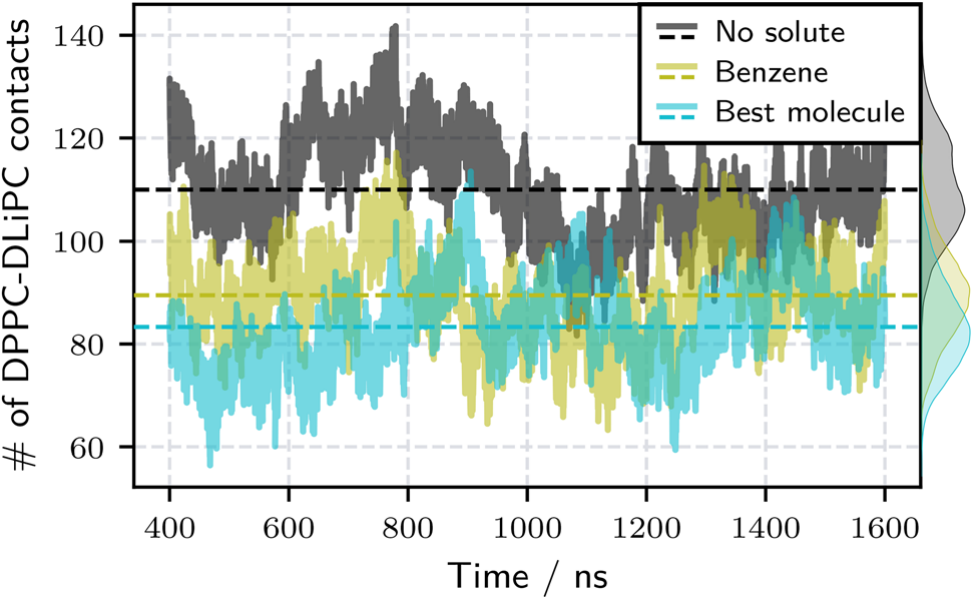
Time evolution of DPPC–DLiPC lipid contacts in ternary bilayers over 1200 ns CG MD simulations (excluding 400 ns for equilibration). Three conditions are compared: a bilayer without solutes (black), a bilayer containing benzene as a known demixing agent (olive green), and one with the top-performing optimized molecule from Fig. 6 (cyan), each at a solute/lipid mass ratio of 4.8%. Dashed horizontal lines indicate mean contact numbers. The optimized molecule reduces DPPC–DLiPC contacts more than benzene, demonstrating a stronger phase-separation effect.

Directly measuring bilayer phase separation requires significant simulation time and is therefore computationally expensive. Instead, we estimated demixing effects from free-energy differences. To validate this approach and confirm that the identified candidates indeed promote phase separation, we perform 1600 ns MD simulations (in reduced CG units) of the best candidate (top left in Fig. 6) in a ternary lipid bilayer system. Using this method to evaluate the demixing effect required one to two orders of magnitude more wall time than the free energy-based scoring used for the optimization. As a reference, we employ benzene, previously identified by Barnoud *et al.*^32^ as a potent driver of lipid bilayer phase separation. Following their protocol, we use a solute/lipid mass ratio of 4.8% (see Section S2.5 of the SI for composition details). Phase separation was quantified by tracking DLiPC and DPPC contacts over the simulation trajectory. Fig. 7 presents the evolution of these contacts throughout the simulation, with dashed lines indicating average values. Each trajectory's initial 400 ns were discarded to ensure equilibration. Additionally, a control simulation without any added solute was conducted. Compared to this bilayer without solutes, our best candidate substantially reduced DLiPC–DPPC contacts, indicating a pronounced effect on bilayer demixing. Our best candidate also outperforms benzene, producing a greater reduction in the number of contacts, suggesting a stronger influence on phospholipid phase separation.

**Fig. 8:**
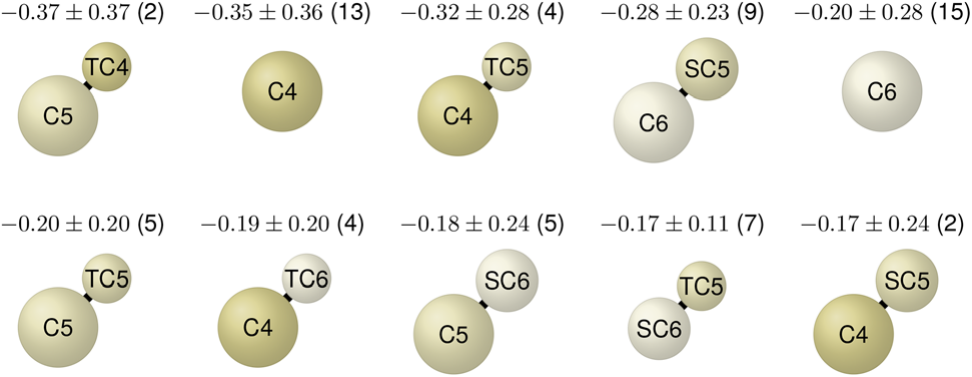
Top ten most influential molecular features contributing to lipid bilayer phase separation, identified *via* LASSO regression of the optimized CG molecules with ΔΔ*G* < 0. Features were limited to single beads and bead pairs. Each panel displays a feature's structure along with its corresponding regression coefficient, bootstrapped uncertainty, and frequency of occurrence within the dataset (number in parentheses). Features only involve hydrophobic C4, C5, and C6 beads and pairs of differing bead sizes.

To identify relevant molecular features and design rules from the set of optimized molecules, we applied LASSO regression analogous to Mohr *et al.*^26^ Derived rules could subsequently inform the design of atomistic structures. We analyzed single-bead and bead-pair features across all molecules with ΔΔ*G* < 0, yielding 85 features. Higher-order features were not included due to the size of the dataset. Feature extraction and LASSO regression details are provided in Mohr *et al.*^26^ The top ten most relevant molecular features, along with their regression coefficients, bootstrapped uncertainties, and frequencies of occurrence, are shown in Fig. 8. Consistent with our earlier analysis of the top eight molecules, the most influential features involve hydrophobic C4, C5, and C6 beads. Moreover, combinations of a regular-sized and tiny or small-sized bead (indicated by T or S) appear relevant. These derived features provide interpretable insights into the physical interaction mechanisms that drive bilayer phase separation. They can be used to design atomistic molecular structures that exhibit the same phase separation behavior.

### 3.3 Comparison with standard BO

**Fig. 9:**
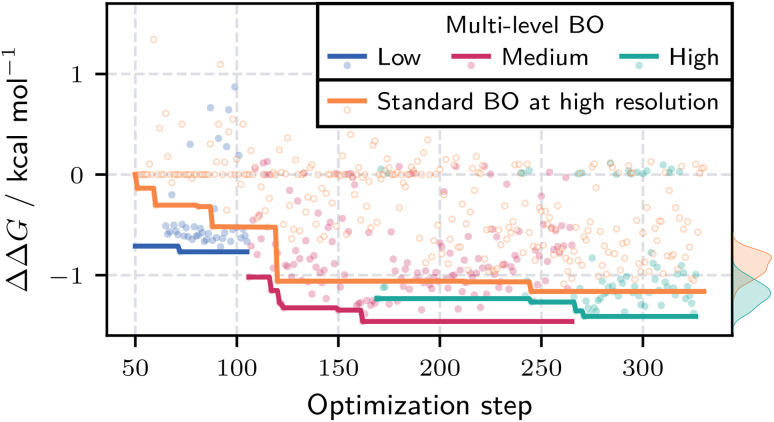
Progression of the ΔΔ*G* values during multi-level and standard BO runs. Multi-level BO uses evaluations at low (blue), medium (magenta), and high (green) resolutions, while the standard BO (orange) operates only at the high resolution. Solid lines show the current best value during the optimization. Initialization points are excluded. The multi-level case accounts for the 15 extra evaluations for the low-resolution prior. Kernel density estimates (right edge) reflect the distribution of best 50 high resolution candidates. Multi-level BO consistently achieves lower ΔΔ*G* values, as indicated by the shifted distribution.

Is multi-level BO computationally advantageous compared to BO using only the high-resolution model? To address this, we performed standard BO with the same number of initial points and total evaluations as in the multi-level case. While BO is typically benchmarked by averaging the cumulative best result across multiple runs to reduce initialization bias, this is computationally infeasible for our bilayer demixing system. Instead, we compare the distributions of obtained ΔΔ*G* values and the cumulative best result within single runs. We provide a toy model comparison of results averaged over multiple runs in Section S2.7 of the SI. Fig. 9 presents the progression of the best ΔΔ*G* values for both optimization approaches. The diagram excludes the 50 initialization points and accounts for the 15 additional evaluations required to construct the low-resolution prior for the multi-level approach. The multi-level BO consistently outperforms the standard BO, achieving superior cumulative best values (solid lines) across all resolution levels. Additionally, the distribution (based on the best 50 molecules) and scatter plots in orange and green highlight that multi-level BO not only finds a better overall candidate, but multiple candidates with significantly lower ΔΔ*G* values than the standard BO optimization. The peak of the multi-level BO distribution is shifted toward lower ΔΔ*G* values compared to the standard BO optimization.

### 3.4 Chemical neighborhood sizes across resolutions

Our multi-level BO algorithm relies on the assumption that the free-energy landscape over the learned chemical representations is smoother at lower resolutions. To test this, we introduce the concept of chemical neighborhoods and analyze their sizes across different resolution levels. We define a chemical neighborhood as a region in chemical space containing similar molecules. Similarity implies that known properties about one molecule help predict properties of its neighbors. Here, neighborhood size is determined by the lengthscale *ξ*_*l*_ of an RBF kernel fitted in a GP regression. This length scale quantifies correlations between points in the latent space and is thus an intrinsic measure of chemical neighborhood size. To obtain the *ξ*_*l*_, we fit independent GP models to the evaluated molecules at each resolution level, excluding lower-resolution priors to prevent bias. Neighborhood size is then calculated as the average number of neighbors within a distance *d*, where *d* = *αξ*_*l*_ and *α* = 0.5 determines the required similarity for a chemical neighborhood.

**Fig. 10:**
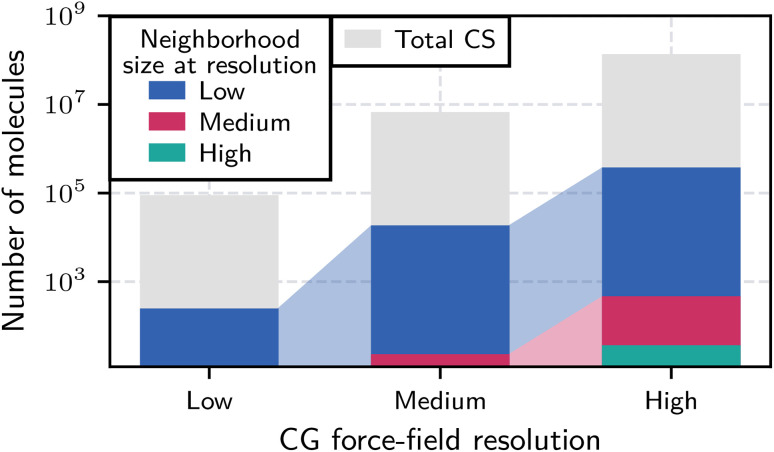
Chemical neighborhood sizes across different CG resolutions of CS. The chart shows the number of molecules within a chemical neighborhood at low (blue), medium (pink), and high (teal) resolution, derived by fitting the lengthscale of a GP RBF kernel to the evaluated molecule data. The total number of molecules is shown in gray. Lower-resolution neighborhoods are mapped to higher resolutions by considering average molecule densities. These different neighborhood sizes reflect the varying smoothness of the free-energy landscape across different CS resolutions.

Fig. 10 shows relationships between the obtained neighborhood sizes, the total number of molecules in the chemical space, and neighborhoods from lower resolutions mapped to higher resolutions (exact numbers in Section S2.6 of the SI). Considering the logarithmic scale of the *y*-axis, we observe that neighborhood sizes span several orders of magnitude across the three resolutions. When mapped to medium or high resolution, low-resolution neighborhoods with about 249 molecules expand to about 18 600 and 378 000 molecules. Similarly, a medium-resolution neighborhood with about 23 molecules maps to a neighborhood of 468 molecules at high resolution. This exponential scaling suggests that prior information for many high-resolution molecules can be inferred from relatively few low-resolution evaluations. Section S2.8 of the SI further illustrates this by showing the coverage of the higher-resolution latent spaces by mapping evaluated molecules from the lower resolutions. These results support our assumption of a smoother free-energy landscape at lower resolutions.

## 4 Conclusions

This work introduces a multi-level Bayesian optimization (BO) framework for efficient exploration of chemical space (CS). Our method employs multiple levels of coarse-graining to exploit the varying smoothness of free-energy landscapes across different model resolutions. By informing the optimization process at higher resolutions with prior knowledge from lower resolutions, we accelerate the search for optimal molecules. Our BO-based algorithm combines information from multiple resolutions in a Bayesian manner, enabling a funnel-like optimization process through CS. This approach allows us to bypass irrelevant regions of CS at higher-resolution representations, substantially reducing the number of required molecule evaluations and the overall computational cost. We demonstrate the effectiveness of our method by identifying small coarse-grained (CG) molecules that enhance phase separation in a ternary lipid bilayer. Despite evaluating only approximately 3 × 10^−4^% of the total number of high-resolution molecules and assuming no prior knowledge of relevant CS regions, we successfully identified several candidates with a significant impact on lipid bilayer phase separation. Our multi-level approach outperforms standard BO, achieving a better overall best result and obtaining a significantly shifted distribution of evaluated molecules toward stronger effects on phase separation. The optimized CG molecules enable us to extract relevant molecular features and design rules. Our analysis of chemical neighborhood sizes at different resolutions confirms the assumption of smoother free-energy landscapes at lower resolutions. Notably, obtained neighborhood sizes vary by several orders of magnitude, allowing us to get prior information for many molecules at high resolution from a small number of evaluations at low resolution.

In this study, we limited our funnel optimization to the CG level and thus did not derive atomistic structures for the identified candidates. Similar to Mohr *et al.*, atomistic structures could be reconstructed based on the extracted molecular features.^26^ Notably, these features provide an intuitive and interpretable summary of the key chemical factors, providing valuable insight into the underlying physical interaction mechanisms. Moreover, the atomistic resolution could be integrated directly into our multi-level optimization framework. Since each CG bead maps to 10^2^ to 10^4^ atomistic fragments,^54^ the atomistic chemical space is vastly larger. Combined with evaluation costs two to three orders of magnitude higher,^65,66^ this poses challenges. Nevertheless, these cost differences enable approaches like multi-fidelity BO,^27,30^ and high-resolution CG results generally provide an efficient starting point that reduces the number of required atomistic evaluations.

A limitation of our multi-level BO method is its reliance on a hierarchical relationship between resolutions, with higher resolutions required to exhibit sufficient complexity. Although multi-level BO improves efficiency over standard BO for complex optimization landscapes, it may underperform on simpler problems. In our application, the target function—mapping the learned latent representation of CS to free energy—is sufficiently complex and non-smooth to benefit from the multi-level BO strategy. Further work is needed to identify optimal complexity hierarchies and resolution-level differences, which could further enhance efficiency. Another limitation is the increased complexity in implementation and hyperparameter tuning. Multi-level BO requires setting hyperparameters for each resolution, as well as additional parameters for resolution switching. Nevertheless, these hyperparameters are primarily related to the chemical space and can thus be transferred across different molecular optimization tasks.

Beyond its demonstrated application in lipid bilayer phase separation, our multi-level BO framework can solve other optimization problems characterized by free-energy differences. We expect our method to be particularly advantageous in applications with little prior knowledge or training data. Furthermore, integrating our method with a FAIR^67^ data infrastructure and automated simulation workflows, such as Martignac,^68^ will enhance data management, reproducibility, and end-to-end automation, thereby making the multi-level BO approach more systematic and streamlined.

Our work provides a versatile and efficient molecular design and optimization framework, offering a promising direction for tackling complex chemical search problems.

## Author contributions

Luis J. Walter: conceptualization, methodology, software, validation, formal analysis, investigation, writing (original draft, writing – review & editing), visualization, Tristan Bereau: conceptualization, resources, writing (review & editing), supervision, project administration, funding acquisition.

## Conflicts of interest

There are no conflicts to declare.

## Supplementary Material

SC-016-D5SC03855C-s001

## Data Availability

The code for the multi-level Bayesian optimization workflow, the simulation setup, the analysis, and the autoencoder training, as well as the autoencoder models and free-energy results, can be found at https://github.com/BereauLab/Multi-Level-BO-w-Hierarchical-CG. A representative subset of the simulation data is available on NOMAD at https://doi.org/10.17172/NOMAD/2025.05.27-1. We also provide a tutorial showcasing the main concepts of this paper through a simple two-bead molecule optimization: https://github.com/BereauLab/Molecule-Optimization-w-Hierarchical-CG-Tutorial. The supplementary information provides extended methodological details and additional results that support the main findings. See DOI: https://doi.org/10.1039/d5sc03855c.
