# Supplementary material for: Navigating chemical space: multi-level Bayesian optimization with hierarchical coarse-graining
Source: Chem Sci. 2025 Jul 30;16(35):16027–38. doi: 10.1039/d5sc03855c (PMC12332539; doi:10.1039/d5sc03855c)
Supplement: SC-016-D5SC03855C-s001 [file SC-016-D5SC03855C-s001.pdf]

Supplementary Information (SI) for Chemical Science.
This journal is © The Royal Society of Chemistry 2025

Supplementary Information for

Navigating Chemical Space: Multi-Level Bayesian Optimization with Hierarchical Coarse-Graining

Luis J. Walter
Institute for Theoretical Physics, Heidelberg University, 69120 Heidelberg, Germany

Tristan Bereau*
Institute for Theoretical Physics and Interdisciplinary Center for Scientific Computing (IWR), Heidelberg University, 69120 Heidelberg, Germany

1 METHOD RELATED DETAILS

S1.1 Derivation of Lower Resolution Coarse-Grained Models

For our multi-level Bayesian optimization (BO) approach, we define several levels of coarse-grained (CG) resolution. We apply the same spatial coarse-graining scheme—mapping atoms to beads—but achieve different resolutions by varying the number of transferable bead types. Each resolution spans the same chemical space (CS) region but at a different level of chemical detail. As our high-resolution model, we use the Martini3 force field,[1] excluding bead labels and water and divalent ion beads. This high-resolution model includes five Q-beads, six P-beads, six N-beads, six C-beads, and four X-beads for each of the three bead sizes. Because Q-beads carry either a positive or negative charge, we obtain 32 classes per bead size and 96 bead types in total. A detailed description of the bead properties can be found in Souza *et al.*[1] Within our framework, the set of available bead types fully determines a CS resolution. All small molecules that can be assembled from these bead types are part of the corresponding CS resolution (see Section S1.2). We hierarchically combine bead types to construct lower-resolution CG models. Figure 1.1 illustrates the hierarchical relationships among bead types for a single bead size; the same scheme applies across all bead sizes and for both positive and negative Q-bead charges.

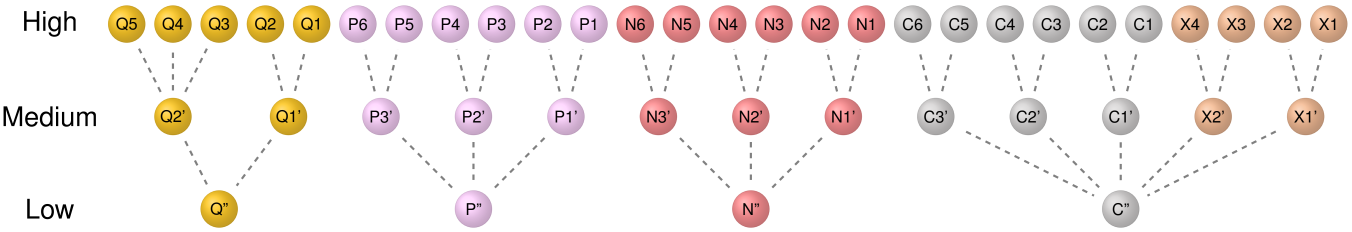

FIG. 1.1. Bead hierarchy for the three coarse-grained (CG) resolutions. The high-resolution model, which is based on the Martini3 force field,[1] contains 27 distinct bead classes per bead size; accounting for charges on the Q-beads increases this to 32 classes per bead size. The dashed lines indicate how bead types are grouped into medium- and low-resolution types.

The Martini3 model defines only general non-bonded interactions; intra-molecular interactions must be parameterized individually for each molecule. While charged interactions are described using a standard electrostatic potential, the parameterized Lennard-Jones interactions are specific to Martini3. Because our high-resolution model is based on Martini3, we could directly adopt its parameters in our simulations. Lennard-Jones parameters for the lower-resolution models were obtained by averaging the corresponding high-resolution parameters. For example, the parameters for the P" bead were calculated by averaging over the P1 to P6 beads in the Martini3 model. Since bead sizes remain the same across resolutions, only the interaction strength of the Lennard-Jones potential (denoted as ϵ) is effectively averaged.[2] Figure 1.2 shows the relative standard deviation of the averaged ϵ parameters for both the medium- and low-resolution models. At the low and medium resolutions, the relative standard deviation is below 10% for approximately 67% and 94% of the parameters, respectively. These relatively slight variations suggest that the combination of bead types is reasonable. As expected, the low-resolution model exhibits greater variability in the averaged parameters.

* bereau@uni-heidelberg.de

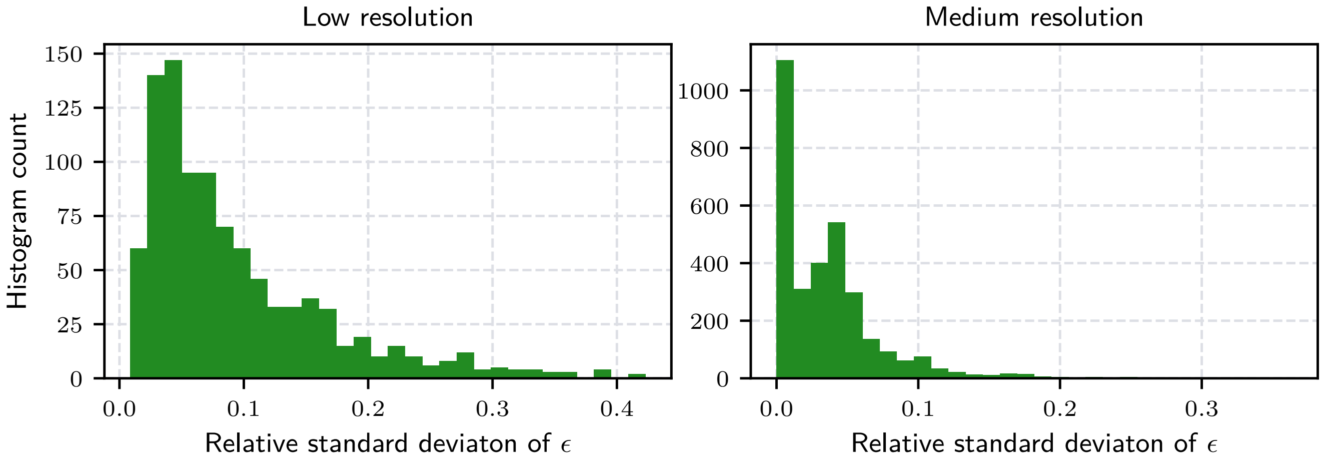

FIG. 1.2. Relative standard deviations of the Lennard-Jones parameter ϵ^2 from the parameter averaging for the medium- and low-resolution CG models. Variations in the averaged low-resolution parameters are higher, as expected by the broader range of bead types.

S1.2 Molecular Graph Enumeration

Since Martini3 only characterizes non-bonded interactions between beads, intra-atomic bonded forces must be determined individually for each molecule. However, starting from atomistic structures and mapping them to CG representations is not feasible across large regions of chemical space. Therefore, we generated molecules directly at the CG level, which has fewer combinatorial possibilities due to the reduced resolution. Although we bypassed the atomistic level initially, backmapping remained possible based on the properties of the beads.[3]

To enumerate CG small-molecule CS, we applied several simplifications and assumptions. We treated small molecules as graphs with fixed, bead-size-dependent bond lengths (listed in Table 1.1). Furthermore, we neglected angle and dihedral interactions, a choice justified by the small size of the molecules. While using fixed bond lengths ignored some chemical details, we assumed that the bead identities and their overall arrangement were more critical than minor variations in bond lengths. We further restricted the small-molecule CS to molecules containing up to four CG beads, corresponding to atomistic molecules with up to 16 heavy atoms.

The main challenge in enumerating all graphs with a given number of nodes was avoiding duplicates, i.e., isomorphic graphs. To address this, we separated bead-type enumeration from bond/topology generation. Enumerating unique sets of bead types effectively reduced to a combination-with-repetition problem, which was straightforward to solve using standard tools, such as those available in Python.[4] Since many bead-type combinations shared the same unique bond configurations, we could reuse generated topologies across multiple bead-type sets. For example, the bead sets {C1, C1, C2, C3} and {C2, C2, C1, C4} shared the same set of bond configurations.

The generation of topologies for a given set of bead types corresponds to generating all non-isomorphic graphs. We employed a two-step filtering strategy to avoid computationally infeasible exhaustive graph isomorphism checks. First, we enumerated all possible topologies using an adjacency matrix representation. To check whether a graph had been previously generated, we created a simple vectorial fingerprint based on node degrees and the number of bonds per bead type. This fingerprint was identical for isomorphic graphs but not necessarily unique for non-isomorphic ones. By storing previously generated graphs in a dictionary-like structure keyed by these fingerprints, we restricted expensive graph isomorphism checks to graphs sharing the same fingerprint.

TABLE 1.1. Bead-size–dependent bond lengths. These bond lengths are used to convert molecular graphs into CG representations suitable for simulation. R, S, and T correspond to regular, small, and tiny beads.

Bead size 1	Bead size 2	Bond length / nm
T	T	0.29
T	S	0.31
S	S	0.33
T	R	0.33
S	R	0.35
R	R	0.38

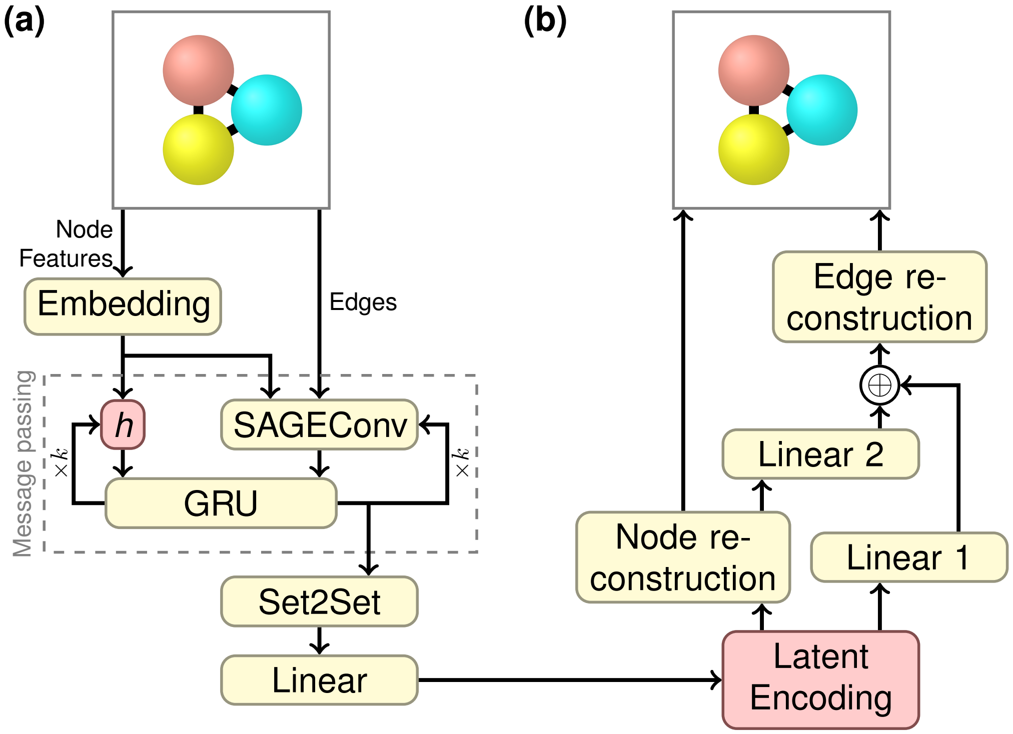

FIG. 1.3. Overview of our regularized autoencoder (RAE) architecture. (a) The molecular graph encoder includes a node embedding layer, a SAGEConv- and GRU-based[5–7] message passing, and Set2Set[8] aggregation. (b) The decoder comprises four feedforward neural networks (FNNs) that sequentially reconstruct the node features and then the adjacency matrix, using the reconstructed nodes and the latent encoding. Although the decoder is not permutation invariant, the loss function is. The symbol $\oplus$ denotes concatenation. Parameter details are listed in Table 1.2. The architecture is inspired by Mohr *et al.*[3]

Overall, the separation of bead and bond generation, the reuse of bond configurations across equivalent bead sets, and the two-stage isomorphism check allowed us to generate unique molecular topologies efficiently. Although we focused on molecules with up to four beads, the molecule generation algorithm could enumerate much larger molecular graphs.

The exact numbers of generated molecular graphs with up to four beads for the three CS resolutions with 15, 45, and 96 available bead types were 89960, 6742680, and 136870880, respectively.

S1.3 Autoencoder for Molecular Graph Embedding

We encoded all previously enumerated molecules into a five-dimensional latent representation to facilitate molecular optimization. This representation enabled distance calculations between molecules. Assuming a sufficiently smooth molecular encoding, this distance was used to measure molecular similarity. We employed a graph neural network (GNN)-based regularized autoencoder (RAE)[9] model to generate a latent encoding for each CS resolution separately. The general RAE structure was identical for each resolution. However, we used different numbers of learnable parameters, with autoencoders for higher CS resolutions containing more parameters. We implemented the autoencoder using the PyTorch and PyTorchGeometric libraries.[10,11] The general architecture was inspired by Mohr *et al.*[3]

The encoder part of the autoencoder consisted of a SAGEConv- and GRU-based[5–7] GNN with $k = 4$ message-passing steps. The GNN-based architecture ensured a permutation-invariant encoding. Figure 1.3a illustrates the architecture of the graph encoder, and Table 1.2 lists the hyperparameters for each component.

The decoder part of the autoencoder consisted of four feedforward neural networks (FNNs). First, the node features were decoded from the latent space representation. Next, the decoded nodes were passed through another FNN and concatenated with an additional decoded representation of the latent space. Finally, the concatenated result was passed through a final FNN to generate the triangular part of the symmetric adjacency matrix of the graph. Figure 1.3b shows the graph decoder architecture, and Table 1.2 provides the corresponding hyperparameters. Since the nodes and adjacency matrix were decoded each in a single step, the decoder was not permutation invariant. However, we observed better autoencoder performance with a one-shot decoding than permutation-invariant decoding strategies.

For training, we used an Adam optimizer[13] with the standard parameters $\beta_1 = 0.9$ and $\beta_2 = 0.999$ and a learning

TABLE 1.2. Architecture and parameter details for the RAE molecular encoder. "Layer name" refers to the labels shown in Figure 1.3, and "Model" describes the corresponding applied function. x denotes the input to each layer, and α corresponds to the LeakyReLU activation function.[12] All linear layers included a bias term. The third to fifth columns report the number of parameters for the linear transformations, excluding bias terms. When all three resolutions used the same transformation, values are provided only once for clarity.

		**Parameters for Encoding at Resolution**		
**Layer Name**	**Model**	**Low**	**Medium**	**High**
Embedding	$\alpha(W_2\ \alpha(W_1\ x))$	$W_1 : 10 \times 128$<br>$W_2 : 128 \times 64$	$W_1 : 19 \times 256$<br>$W_2 : 256 \times 64$	$W_1 : 33 \times 512$<br>$W_2 : 512 \times 64$
SAGEConv	$W_1\ x_i + W_2\ \text{mean}_{j \in \mathcal{N}(i)} x_j$		$W_1 : 64 \times 64$<br>$W_2 : 64 \times 64$	
GRU	GRU(x, h)		dim. 64	
Linear	$\alpha(W_2\ \alpha(W_1 x))$		$W_1 : 128 \times 64$<br>$W_2 : 64 \times 5$	
Node reconstruction	$W_3\ \alpha(W_2\ \alpha(W_1 x))$	$W_1 : 5 \times 1024$<br>$W_2 : 1024 \times 512$<br>$W_3 : 512 \times 44$	$W_1 : 5 \times 1024$<br>$W_2 : 1024 \times 1024$<br>$W_3 : 1024 \times 80$	$W_1 : 5 \times 2048$<br>$W_2 : 2048 \times 1024$<br>$W_3 : 1024 \times 136$
Linear 1	$W_2\ \alpha(W_1 x)$		$W_1 : 5 \times 512$<br>$W_2 : 512 \times 512$	
Linear 2	$W_2\ \alpha(W_1 x)$	$W_1 : 10 \times 128$<br>$W_2 : 128 \times 32$	$W_1 : 19 \times 256$<br>$W_2 : 256 \times 32$	$W_1 : 33 \times 512$<br>$W_2 : 512 \times 32$
Edge reconstruction	$W_3\ \alpha(W_2\ \alpha(W_1 x))$		$W_1 : 640 \times 512$<br>$W_2 : 512 \times 512$<br>$W_3 : 512 \times 6$	

TABLE 1.3. Reconstruction accuracies for edges, bead classes, sizes, and charges across all three encoded CS resolutions. For the low-resolution model, accuracies were evaluated over all molecules, while for the medium- and high-resolution models, they were computed on samples of 10^6 molecules.

**Resolution level**	**Reconstruction accuracies**			
	**Edges**	**Bead classes**	**Bead sizes**	**Bead charges**
Low	0.976	0.989	0.993	1.000
Medium	0.994	0.999	0.991	1.000
High	0.987	0.979	0.986	0.998

rate of 1×10^{-3}. The loss function

$$\mathcal{L} = \mathcal{L}_{\text{reconstruction}} + \lambda_{\text{weights}} \mathcal{L}_{w-\text{decoder}} + \lambda_{\text{latent}} \|z\|_2 \tag{1}$$

consisted of a reconstruction term $\mathcal{L}_{\text{reconstruction}}$, a decoder weight regularization term $\mathcal{L}_{w-\text{decoder}}$ with prefactor $\lambda_{\text{weights}} = 1 \times 10^{-5}$, and a L2-regularization of the latent space z with prefactor $\lambda_{\text{latent}} = 1 \times 10^{-4}$. The reconstruction loss $\mathcal{L}_{\text{reconstruction}}$ consisted of three cross-entropy loss terms for the categorical node features, a mean squared error term for the octanol-water partitioning free energy, and a binary cross-entropy for the triangular adjacency matrix. We minimized $\mathcal{L}_{\text{reconstruction}}$ over all possible graph permutations to achieve a permutation-invariant reconstruction loss. Because we considered only graphs with up to four beads, evaluating all permutations was computationally feasible. For larger molecules, however, this approach becomes prohibitively expensive. We used a training batch size of 16,384 molecular graphs. The node features included three categorical properties—class, size, and charge—and the octanol-water partitioning free energy as a continuous property. All categorical features were one-hot encoded and concatenated with the octanol-water partitioning free energy before encoding. We used a dummy bead class during node reconstruction to reconstruct molecules with fewer than four beads. If this dummy class was predicted, the node and its corresponding edges were ignored.

Because our goal was to obtain a numerical encoding of the chemical space without generating new molecules, we discarded the decoder after training. Nonetheless, we aimed for high reconstruction accuracy, indicating high information content in the encoded representation. Table 1.3 reports the reconstruction accuracies for all three CS encodings.

S1.4 Mapping Between Latent Spaces and Neighborhood Calculations

In our multi-level BO algorithm, we used lower-resolution information as a prior to guide exploration at higher resolutions of CS, which required a mapping between resolutions. Because we learned the CS representations separately for each CG resolution, the latent vectors could not be directly transferred across resolutions. However, since we defined a known bead-type mapping between resolutions (see Section S1.1), we could establish a correspondence between molecules at each resolution and therefore between their latent representations. For example, the two-bead molecule P4–C5 at high resolution could be mapped to P2'–C3' at medium resolution and to P"–C" at low resolution.

In practice, we stored all enumerated molecules in an indexable database, along with their latent representation and a reference to the corresponding next-lower-resolution molecule. Creating a database index for this reference at both resolution levels made it possible to map molecules quickly between lower and higher resolutions. As a result, information from lower-resolution Gaussian process (GP) models could be easily transferred to higher-resolution levels.

For the optimization at higher resolutions, our multi-level BO algorithm focused on latent space regions with the highest likelihood of favorable $\Delta\Delta G$ values. In each optimization iteration, the expected improvement (EI) based on the GP model was maximized only within the neighborhoods of the most promising molecules. This neighborhood-focused maximization at resolution level l (with $l \geq 2$) followed several steps.

First, we selected the top m molecules $X_{l-1,\text{top-}m}$ with the lowest $\Delta\Delta G$ values from the next-lower resolution level $l-1$: $X_{l-1,\text{top-}m} = \arg\max_m \Delta\Delta G(x)$ for $x \in X_{l-1}$. The hyperparameter m, which controlled the balance between optimization accuracy and computational cost, was set to 30.

Next, we identified the set of molecules $\tilde{P}_l$ at level l that corresponded to these m top-ranked molecules at level $l-1$: $\tilde{P}_l = \{x \in \mathcal{X}_l | \mathcal{M}(x) \in X_{l-1,\text{top-}m}\}$. Using a cell list built over the latent space, we then assembled the set P_l of all molecules at level l that were located in the same or an adjacent cell as any molecule in $\tilde{P}_l$: $P_l = \{x \in \mathcal{X}_l | \exists z \in \tilde{P}_l, d(x,z) \leq 1\}$, where $d(x,z)$ was the cell-list-based distance metric.

Because the latent space distribution was approximately Gaussian—due to the L_2 loss applied during autoencoder training—we determined the cell sizes in the cell list based on a Gaussian distribution function. Finally, the EI was maximized over all points $x \in P_l$.

This neighborhood-restricted EI maximization allowed the algorithm to efficiently concentrate on promising regions of chemical space while maintaining computational tractability.

S1.5 Scoring of Water- or Interface-Localizing Molecules

To estimate a molecule's lipid bilayer demixing behavior, we calculated the free-energy difference between inserting the molecule into a ternary versus a pure DLiPC bilayer, following the approach of Centi *et al.*[14] We focused solely on the free-energy difference between the bilayer centers to avoid the computational expense of computing potential of mean force (PMF) profiles in both bilayers. Prior observations that molecules influencing bilayer mixing tend to localize near the bilayer center justified this simplification.[14] Specifically, we computed the free-energy difference $\Delta\Delta G = \Delta G_{\text{center}} - \Delta G_{\text{DLiPC}}$ between positioning the molecule at the center of the ternary and the center of the DLiPC bilayer. However, this measure was meaningful only for molecules localizing at the bilayer center. To account for localization behavior, we combined $\Delta\Delta G$ with a score S that penalized molecules preferring bulk water or the bilayer interface over the center. The penalty score was defined by the following conditional equation:

$$S = \begin{cases} 0.5 + \min(\Delta G_{\text{water}} - \Delta G_{\text{center}}, 25)\dfrac{2}{25}, & \text{if } \Delta G_{\text{water}} > \Delta G_{\text{center}} \\ \min(\Delta G_{\text{interface}} - \Delta G_{\text{center}}, 3)\dfrac{0.5}{3}, & \text{else if } \Delta G_{\text{interface}} > \Delta G_{\text{center}} \\ 0, & \text{else.} \end{cases} \quad (2)$$

This equation scaled the free-energy differences between the center and the interface or water phase to values between $0\,\text{kcal}\,\text{mol}^{-1}$ to $2.5\,\text{kcal}\,\text{mol}^{-1}$. The scaling and thresholds were chosen empirically, informed by typical ΔG_{center}, $\Delta G_{\text{interface}}$, and ΔG_{water} values and by expected $\Delta\Delta G$ patterns reported in prior simulations.[14] The formulation assumed that most $\Delta G_{\text{water}} - \Delta G_{\text{center}}$ values fell between $0\,\text{kcal}\,\text{mol}^{-1}$ to $25\,\text{kcal}\,\text{mol}^{-1}$, and $\Delta G_{\text{interface}} - \Delta G_{\text{center}}$ values between $0\,\text{kcal}\,\text{mol}^{-1}$ to $3\,\text{kcal}\,\text{mol}^{-1}$.

By incorporating the score S, we made the free-energy landscape more meaningful in regions of chemical space where molecules did not localize at the bilayer center. Without this adjustment, raw $\Delta\Delta G$ values would have produced an uninformative landscape over large regions, hindering optimization within CS.

S1.6 Simulation Parameters

Each molecule evaluation within our active learning loop involved up to four thermodynamic integration (TI) calculations. For each of these calculations, we employed 26 or 36 linearly spaced λ-steps for uncharged and charged molecules, respectively. Simulations were conducted using GROMACS[15,16] in an automated high-throughput workflow, applying a consistent configuration across all runs. Initially, each system underwent a setup step, an energy minimization, and an equilibration. The same equilibrated structure was subsequently used for all λ-step simulations. For the system setup, the small CG molecule was placed in the respective environment—either bulk water or one of the lipid bilayer systems. Energy minimization was performed using the steepest descent method with 30,000 optimization steps. For the equilibration, we used an integration time step of 10 fs (in reduced CG units) over 40,000 integration steps. The subsequent λ-step simulations used a 20 fs integration time step with the following number of integration steps, depending on the system:

Bulk water:	400,000
DLiPC bilayer center:	400,000
Ternary bilayer center:	900,000
Ternary bilayer interface:	1,200,000

Different step counts were used to balance computational efficiency with adequate convergence, recognizing, for example, the greater heterogeneity of the ternary bilayer compared to the pure DLiPC bilayer. Due to the conditional construction of the score, simulations at the ternary bilayer interface and the DLiPC center were skipped for molecules that did not insert into the bilayer. As shown in Table 1.4, this results in a lower average computational load per simulation at low resolution than at both higher resolutions. For the λ simulations, we achieved an average simulation performance of 4.3 µs/day or 2.16×10^8 steps/day.

TABLE 1.4. Total number of simulations and simulation steps per resolution level including all equilibration and λ simulations. Due to the conditional construction of the score and funnel-like optimization, lower resolution evaluations have a larger average computational load.

Resolution	# simulations	# total simulation steps
Low	91 (29.2%)	2.0×10^{10} (23.3%)
Medium	148 (47.4%)	4.5×10^{10} (51.4%)
High	73 (23.4%)	2.2×10^{10} (25.3%)

Both the equilibration and λ-step simulations employed a leap-frog stochastic dynamics integrator at a temperature of 305 K, with an inverse friction constant of 2 ps. Pressure was maintained at 1 bar using a semi-isotropic C-rescale barostat[17] with a relaxation time constant of 4 ps and a compressibility of $3 \times 10^{-4}\,\mathrm{bar}^{-1}$. Coulomb interactions beyond a 1.2 nm cutoff were treated using the reaction field method, while Lennard-Jones interactions were truncated and shifted at 1.2 nm. To minimize artifacts, we set the GROMACS parameters `verlet-buffer-tolerance` to -1 and `rlist` to 1.4.[18] To restrain the molecule's z-position within the lipid bilayer, we applied an umbrella potential with a force constant of $500\,\mathrm{kJ\,mol^{-1}\,nm^{-2}}$. The restraining reference was defined over a cylindrical bilayer region with a radius of 1.5 nm around the inserted molecule (`pull-coord1-geometry = cylinder`).

To measure or restrain the number of DPPC-DIPC contacts in the ternary bilayer, we used the colvars module in GROMACS.[19] For each leaflet, a collective variable was defined using the coordination number (`coordNum` option) between the first C1 beads of the two phospholipids, with a cutoff distance of 1.1 nm. To improve performance, we set the colvars pair list `tolerance` to 0.001 and the pair list frequency to 100. The collective variable was restrained using a harmonic potential with a force constant scaling of 1.

2 RESULT RELATED DETAILS

S2.1 Analysis of the Learned Molecular Latent Space Representations

All molecules in the chemical space are mapped to a five-dimensional latent representation to facilitate molecule optimization. Navigation within this continuous latent space is more tractable than in the discrete molecular space. The latent representations are learned independently for each resolution level using a GNN-based RAE (see Section S1.3). Visualizing these five-dimensional latent spaces in an informative manner is inherently challenging. A two-dimensional principal component analysis (PCA) projection provides an overview of the latent structure (see Figure 4 in the main text). However, as all five latent dimensions carry meaningful information, the PCA projection captures only a limited portion of the variance, inevitably discarding essential details. To provide deeper insights into the learned representations, four out of the ten combinatorially possible two-dimensional projections are visualized for each resolution level, colored by various molecular properties. Figure 2.1 shows the distribution of the summed

octanol–water transfer free energies of individual beads, $\Delta\Delta G_{\text{octanol}\rightarrow\text{water}}$, across the latent space. A clear correspondence between the latent space structure and the summed $\Delta\Delta G_{\text{octanol}\rightarrow\text{water}}$ is observed. This behavior is expected given that these values serve as inputs and reconstruction targets for the autoencoder. Additional visualizations for total molecular charge, molecular weight, number of nodes, and number of edges are presented in Figures 2.2, 2.3, 2.4, and 2.5, respectively. For medium- and high-resolution levels, these visualizations are based on random latent space samples of 100,000 molecules. All figures reveal discernible structural patterns associated with the underlying molecular properties.

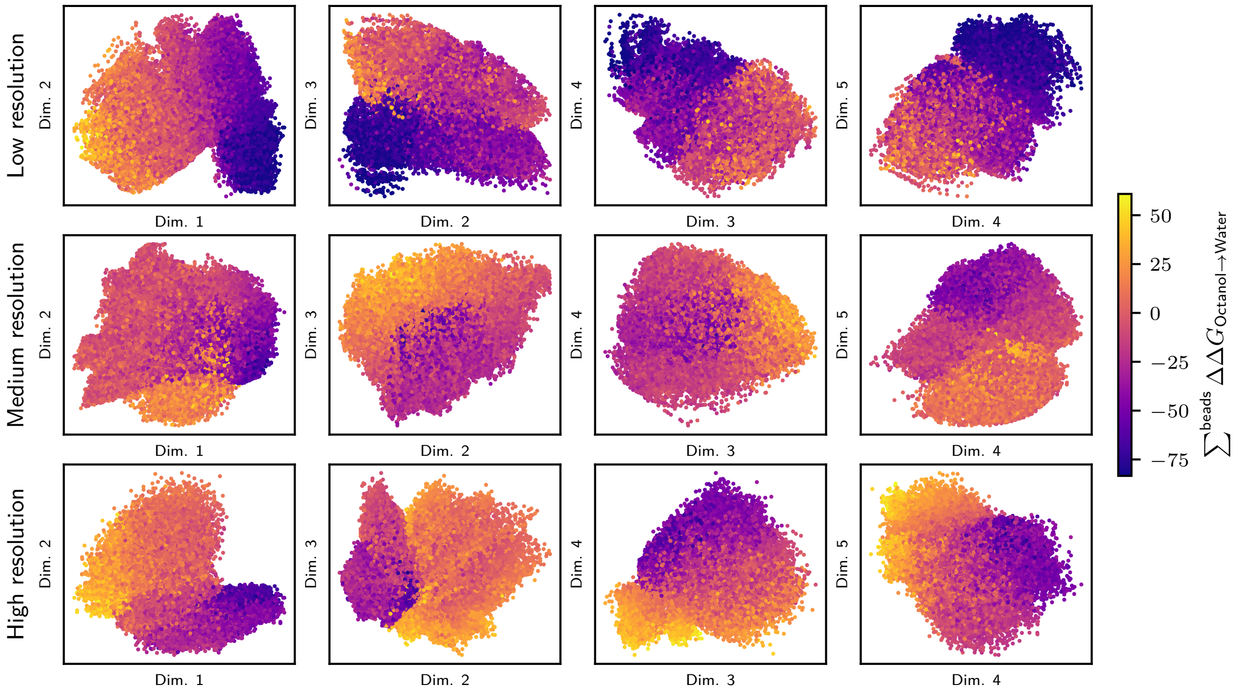

FIG. 2.1. Learned latent spaces for the three resolution levels, with points colored by sum over octanol-water transfer free energies for individual beads. For the medium and high resolution, 100,000 molecules were randomly sampled from the full latent space.

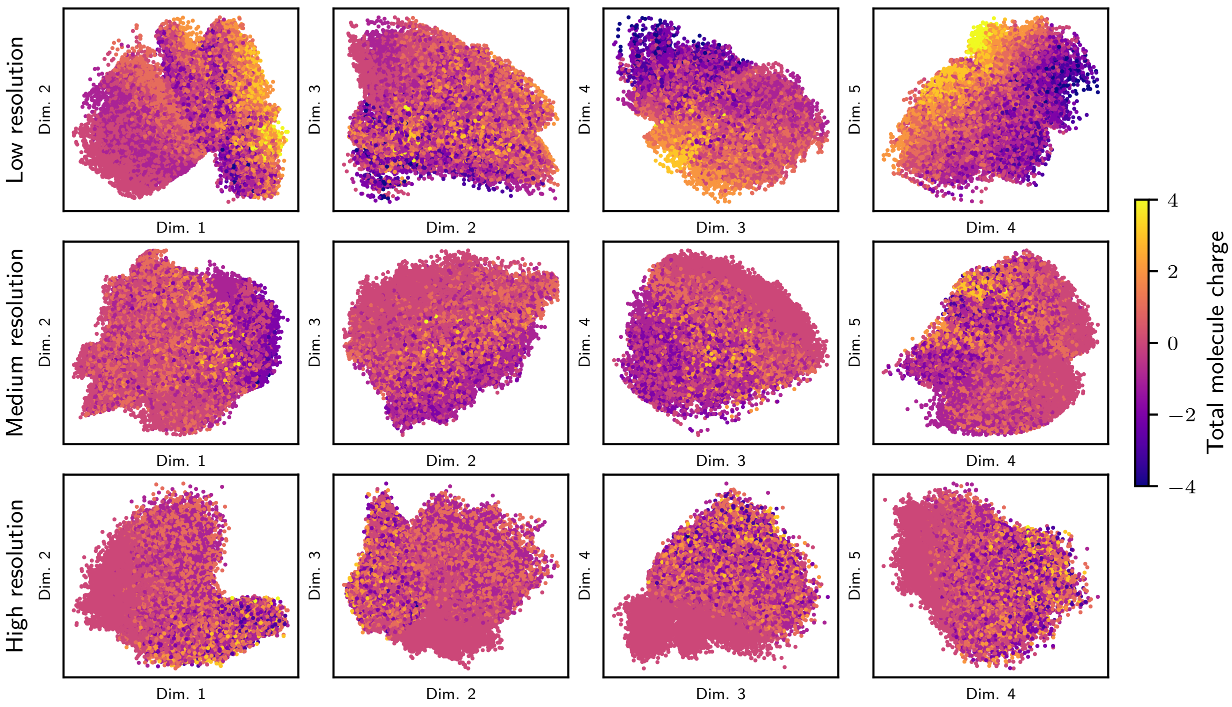

FIG. 2.2. Learned latent spaces for the three resolution levels, with points colored by the total molecule charge. For the medium and high resolution, 100,000 molecules were randomly sampled from the full latent space.

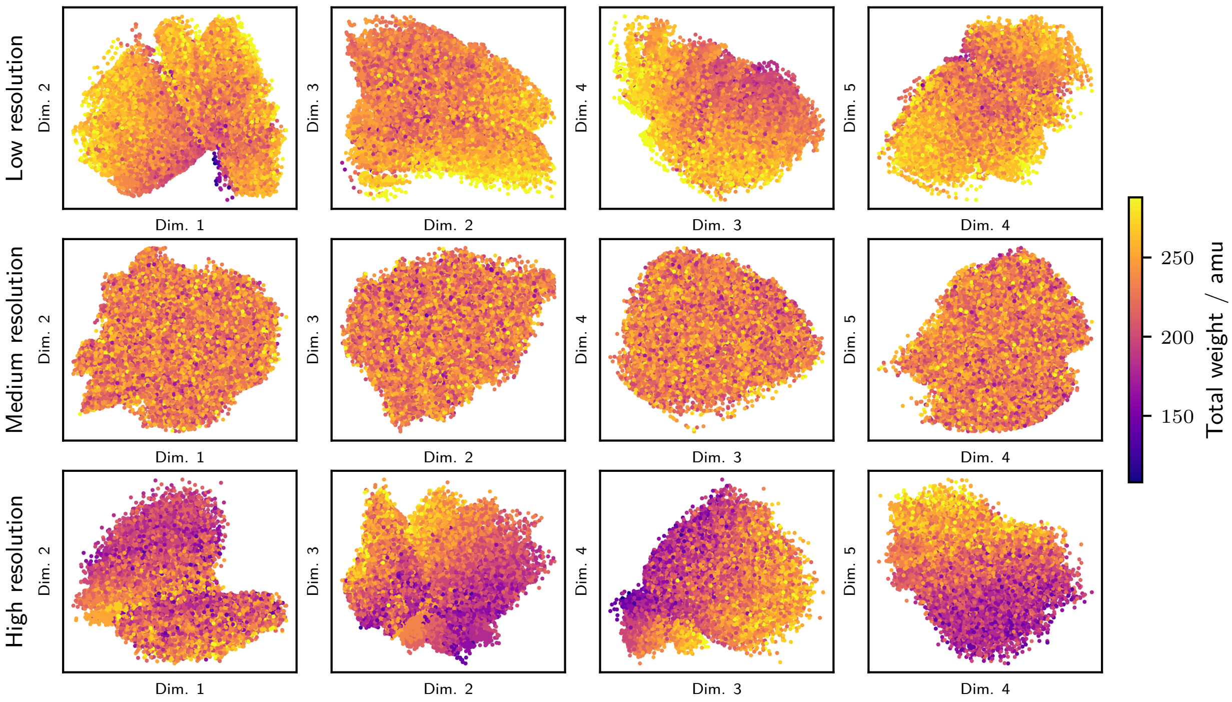

FIG. 2.3. Learned latent spaces for the three resolution levels, with points colored by the total molecule weight. For the medium and high resolution, 100,000 molecules were randomly sampled from the full latent space.

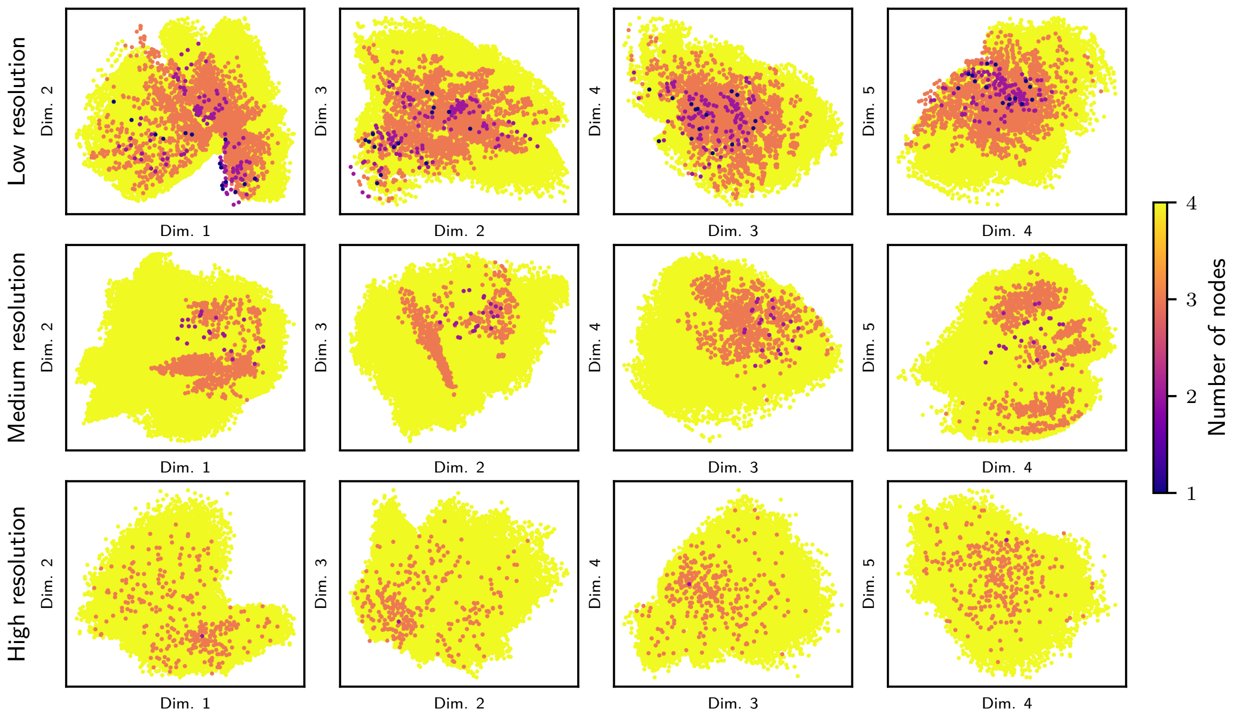

FIG. 2.4. Learned latent spaces for the three resolution levels, with points colored by the number of nodes per molecule. For the medium and high resolution, 100,000 molecules were randomly sampled. Scatter points are drawn in decreasing node count order to enhance the visibility of molecules with low node counts.

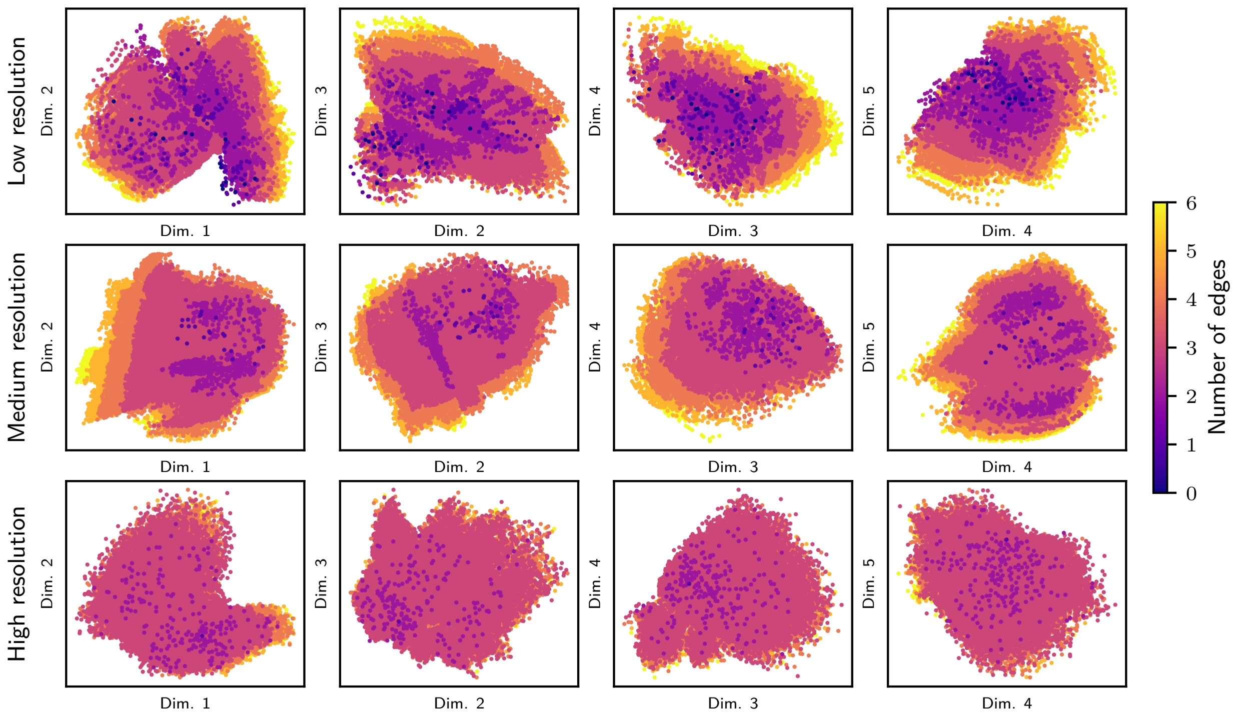

FIG. 2.5. Learned latent spaces for the three resolution levels, with points colored by the number of edges per molecule. For the medium and high resolution, 100,000 molecules were randomly sampled. Scatter points are drawn in decreasing node count order to enhance the visibility of molecules with low node counts.

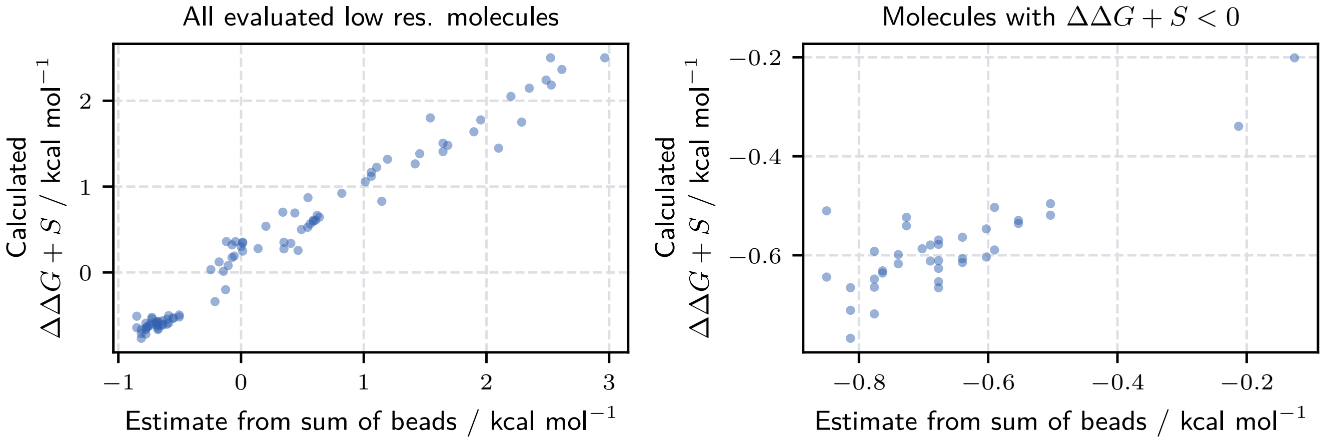

FIG. 2.6. Comparison of calculated molecule $\Delta\Delta G + S$ values with estimates based on a linear combination of the individual bead $\Delta\Delta G + S$ contributions. The left panel presents results for all molecules evaluated at the low-resolution level. Many molecules do not localize near the bilayer center, resulting in positive S values. The right panel includes only molecule results with negative $\Delta\Delta G$ (i.e., $S = 0$). Both panels show a clear correlation between the simulation results and the bead-based estimation.

S2.2 Lowest-Resolution Model Prior

In our multi-level BO algorithm, the GP model at resolution level $l-1$ serves as the mean prior for the GP model at resolution level l. However, no lower-level model is available at the lowest-resolution level ($l = 1$). Rather than using a constant prior, we employ a free-energy estimate based on the additive contribution of individual CG bead free energies:

$$(\widehat{\Delta\Delta G + S})_{\text{molecule}} = \beta \sum^{\text{beads}} (\Delta\Delta G + S)_{\text{bead}}. \tag{3}$$

While correlations between beads influence a molecule's free-energy result, the additivity assumption provides a reasonable approximation. Figure 2.6 compares $\Delta\Delta G + S$ values obtained from full molecule simulations to those estimated by the linear combination of bead contributions. The results show a strong correlation, supporting the validity of the additive assumption as a prior for the GP model. The scaling parameter β was determined by fitting the simulated free energies of the 50 initialization molecules to their corresponding bead sums, yielding $\beta = 0.63$.

S2.3 Evaluation of the $\Delta\Delta G$ Standard Deviation

The GP model with a radial basis function (RBF) kernel (described in Section 2.5 in the main text) has two parameters: the kernel lengthscale, ξ, and the data noise, σ_{n}. Both parameters can be obtained via maximum likelihood estimation during the GP regression process. However, to improve the robustness of the lengthscale estimation, we fixed the value of σ_{n} based on the observed variability in the computed free energies. This was achieved by performing duplicate free-energy calculations for 14 molecules across the four different environments (water, ternary bilayer interface and center, and DLiPC bilayer center), and using the resulting differences to estimate the standard deviations of ΔG, assuming a Gaussian distribution of errors. This approach was also used to determine the number of molecular dynamics (MD) integration steps for each system (see S1.6) required to achieve an acceptable accuracy. Figure 2.7 presents histograms of the observed ΔG differences between paired simulations. Table 2.1 summarizes the corresponding standard deviations. Based on these results and equation 2, we set $\sigma_{\text{n}} = 0.05\,\text{kcal}\,\text{mol}^{-1}$.

TABLE 2.1. Standard deviations of ΔG for different systems.

System	$\sigma_{\Delta G}$ / kcal mol^{-1}
Water	0.056
Ternary bilayer center	0.051
Ternary bilayer interface	0.048
DLiPC bilayer center	0.034

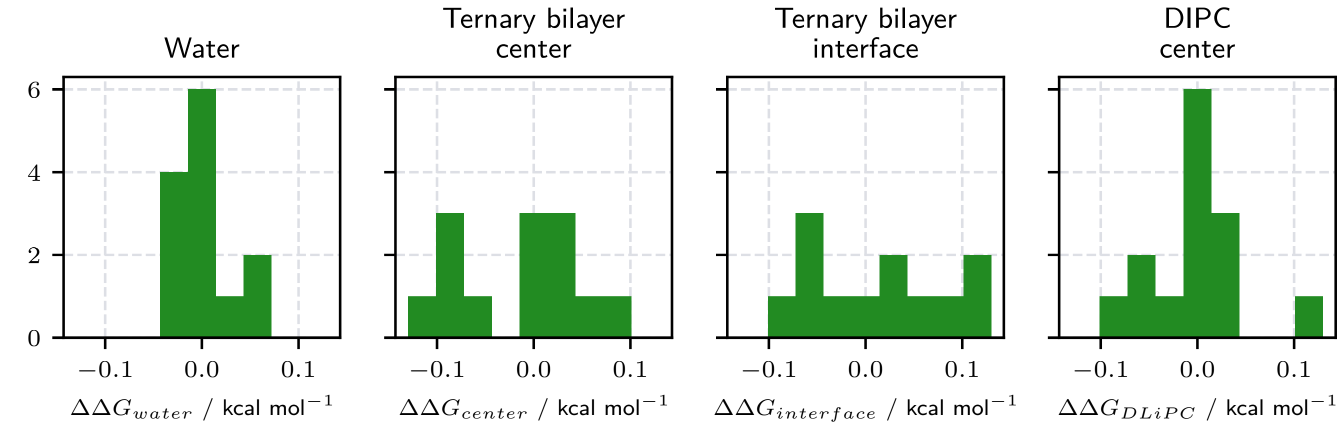

FIG. 2.7. Histograms of differences between two repeated calculations of ΔG values for 14 molecules in water, at the ternary bilayer center and interface, and the DLiPC bilayer center.

S2.4 Best obtained molecules from the low and medium resolution optimization

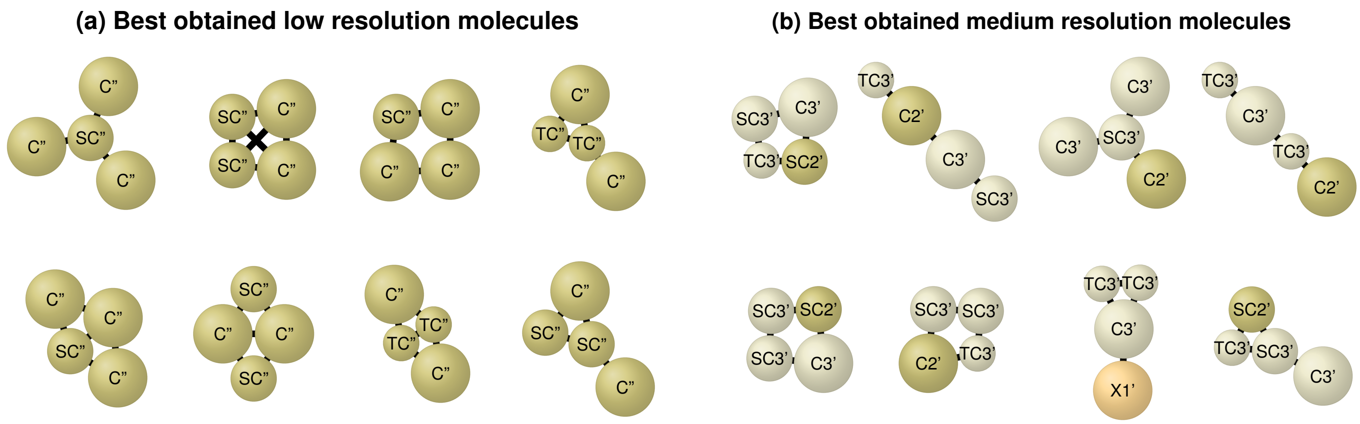

FIG. 2.8. Highest-performing molecules from the (a) low- and (b) medium-resolution models. While the molecules exhibit diverse topologies, they consist solely of C” beads at low resolution, and primarily C2’ and C3’ beads at medium resolution—with one exception involving an X1’ bead. Notably, no C1’ beads are present, despite their mapping to the same C” type at low resolution. Although the low-resolution results already reveal relevant chemical features, incorporating higher-resolution models provides additional chemical detail. According to Barnoud *et al.*[20], the presence of C1’ beads—or their corresponding higher-resolution types C1, C2, and C3—is expected to enhance lipid mixing. This insight is only accessible through the integration of higher-resolution models into the multi-level optimization.

S2.5 System Composition for Direct Demixing Analysis

During the multi-level BO, we do not directly calculate the phospholipid demixing behavior of molecules. Instead, we estimate this behavior based on the free-energy difference of inserting a molecule into a pure DLiPC bilayer versus a ternary bilayer. This approach significantly reduces the computational cost, as converging accurate free-energy differences requires substantially less simulation time than directly observing demixing, which is hampered by significant fluctuations in mixing behavior. However, to validate the predictions from the optimization workflow, it is necessary to assess the phospholipid demixing behavior through direct simulations. For these validation simulations, we increase the bilayer area from $6\,\text{nm}\times 6\,\text{nm}$, as used in the free-energy calculations (see Section 2.6), to $12\,\text{nm}\times 12\,\text{nm}$ to improve statistical reliability. The same DPPC:DLiPC:cholesterol ratio of 7.0:4.7:5.0 is maintained, corresponding to 94 DPPC, 63 DLiPC, and 67 cholesterol molecules. The target molecule is added to the system at a solute-to-lipid mass ratio of 4.8%, calculated using CG masses, to evaluate demixing effects. The total mass of the ternary bilayer is 333 180 Da, derived from $11\times 72\,\text{Da}+1\times 54\,\text{Da} = 846\,\text{Da}$ for DPPC and DLiPC, and $3\times 72\,\text{Da}+4\times 54\,\text{Da}+2\times 36\,\text{Da} = 504\,\text{Da}$ for cholesterol. The best-performing molecule from our multi-level BO has a mass of 234 Da, resulting in 68

molecules being incorporated into the bilayer. For comparison, benzene (108 Da) was added at a total of 148 molecules in its corresponding simulation.

S2.6 Chemical Neighborhood Sizes Based on Gaussian Process Kernel Lengthscales

We introduced the concept of chemical neighborhoods to characterize the smoothness of the learned chemical space representation. A chemical neighborhood is defined as a group of molecules exhibiting similar properties with respect to the optimization target. Larger neighborhoods indicate a smoother free energy or target function landscape across chemical space. We quantified molecular similarity, and consequently the size of a chemical neighborhood, using the lengthscale parameter of an RBF kernel fitted within a GP regression. Smooth functions with lower variability are best modeled with larger lengthscales, while highly variable functions require shorter lengthscales. Thus, larger length scales imply smoother target functions and correspondingly larger chemical neighborhoods. To estimate the lengthscale ξ_l at a given resolution level l, we optimized the negative marginal log likelihood using GPyTorch.[21] The size of a chemical neighborhood was then determined by calculating the average number of neighboring molecules within a latent space distance $d < \alpha\xi_l$, with $\alpha = 0.5$. Smaller values of α imply a stricter similarity criterion, whereas larger values allow for looser similarity within neighborhoods. The average number of neighbors within this distance was computed across ten independent samples of 30,000 randomly selected molecules. The resulting neighborhood sizes, visualized in Figure 10 of the main text, are summarized in Table 2.2. The table also reports the sizes of mapped neighborhoods across different resolution levels. On average, a molecule at low resolution corresponds to approximately 75 molecules at medium resolution, and a molecule at medium resolution corresponds to about 20 molecules at high resolution. These numbers arise from the hierarchical nature of our CG models. The sizes of mapped neighborhoods can be calculated based on these numbers. For example, for the low-resolution neighborhood, we obtain a size of $248.6 \cdot 75 \approx 18,600$ molecules when mapped to the medium resolution.

TABLE 2.2. Chemical neighborhood size results for the three resolution levels. The table includes the average sizes of neighborhoods mapped to higher resolutions (*italic* numbers).

	**Neighborhood size represented at resolution level**		
**Resolution level**	Low	Medium	High
Low	249		
Medium	*18,700*	23	
High	*378,000*	*468*	37

S2.7 Multi-level Bayesian Optimization with a Toy Model

The choice of initialization points can significantly influence the performance of a BO run. Therefore, it is common practice to average results over multiple runs to ensure a statistically meaningful comparison between different BO methods. However, performing multiple optimization runs for our bilayer demixing application is impractical due to the high computational cost of evaluating each molecule. Here, we employed a simple toy model with an easily evaluable molecule score to facilitate a comparative analysis between our multi-level BO algorithm and standard BO at the high-resolution level across multiple runs.

For this toy model, we considered CG-molecule-like objects with exactly two beads ($n \in 1, 2$), each characterized by two properties, b_{np}, that influence the molecule's score. These properties could, for example, represent charge or polarity. As a simplification, we assume a non-permutation-invariant molecule score s given by:

$$s = -0.5(b_{11} - b_{21})^2 + 0.5(b_{11} - 0.3)^2 - b_{12} - (b_{22} - 1)^2 \tag{4}$$

which we aimed to maximize. We defined a discrete set of bead values: $b_{n1} \in \{0.00, 0.33, 0.67, 1.00\}$ and $b_{n2} \in \{0.0, 0.25, 0.5, 0.75, 1.0, 1.25, 1.5, 1.75, 2.0\}$, leading to a total of 1,296 possible molecules in this toy example's chemical space. While direct optimization in this discrete space is relatively simple, we use a learned one-dimensional representation of the chemical space, making the optimization problem more complex and closer to real-world chemical optimizations. For our multi-level optimization, we used a lower-resolution model with fewer discretization steps ($b'_{n1} \in \{0.00, 1.00\}$ and $b'_{n2} \in \{0.0, 1.0, 2.0\}$), reducing the number of possible molecules to 36. Figure 2.9a visualizes the learned one-dimensional representations and corresponding molecule scores. Scatter point colors reflect the correspondence between each low-resolution point and its related high-resolution molecules. Although the scoring function appears relatively simple at low resolution, its structure becomes substantially more complex in the high-resolution model.

We conducted 50 optimization runs using both standard BO and our multi-level BO, following the methodology described in the main text. Each run was initialized with three randomly selected points, followed by 52 optimization

iterations. Where applicable, we utilized the same hyperparameters, like constraints on the BO kernel lengthscale and noise, for both methods. Figure 2.9b shows the average cumulative best result at the high resolution for both optimization approaches. Initially, the multi-level method remains constant due to the optimization occurring at the low-resolution level. As a result, standard BO achieves better performance for a low number of evaluation steps. However, once the multi-level approach transitions to the high-resolution model, it quickly outperforms standard BO on average. Figure 2.9b shows a histogram of the top ten results from each of the 50 optimization runs. The distribution of top molecules found by our multi-level BO is shifted towards higher values and exhibits a sharper peak compared to standard BO. This demonstrates that our method identifies higher cumulative optima and consistently finds multiple solutions near the global optimum. A similar trend was observed in our bilayer demixing application.

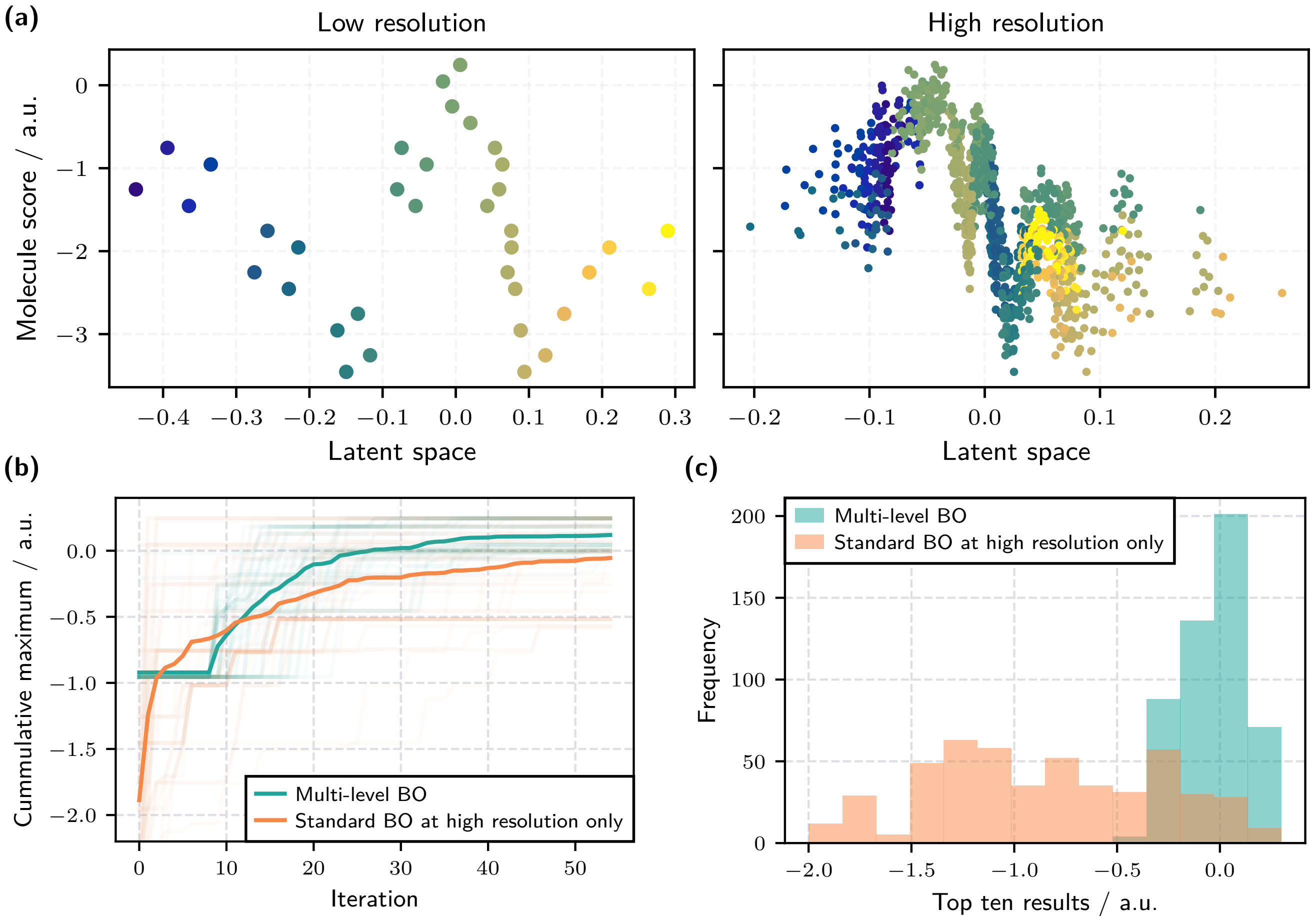

FIG. 2.9. Comparison of standard and multi-level BO on a toy model. (a) Learned latent space representation of the discrete chemical space at low (left) and high resolution (right). Molecule scores (equation 4) are shown on the vertical axis. Colors indicate how low-resolution points correspond to their high-resolution counterparts. The high-resolution landscape is notably more complex than the low-resolution one. (b) Cumulative best high-resolution molecule scores from 50 runs for standard BO (orange) and multi-level BO (teal), with individual runs (shaded) and their averages (solid). The multi-level approach initially plateaus due to the optimization at low resolution (not shown), but quickly surpasses standard BO after switching to the high-resolution model. (c) Histogram of the top ten results per run. Multi-level BO yields a distribution with higher scores and a sharper peak, indicating more consistent convergence toward high-performing solutions compared to standard BO.

S2.8 Mapping of Evaluated Molecules Between Latent Spaces

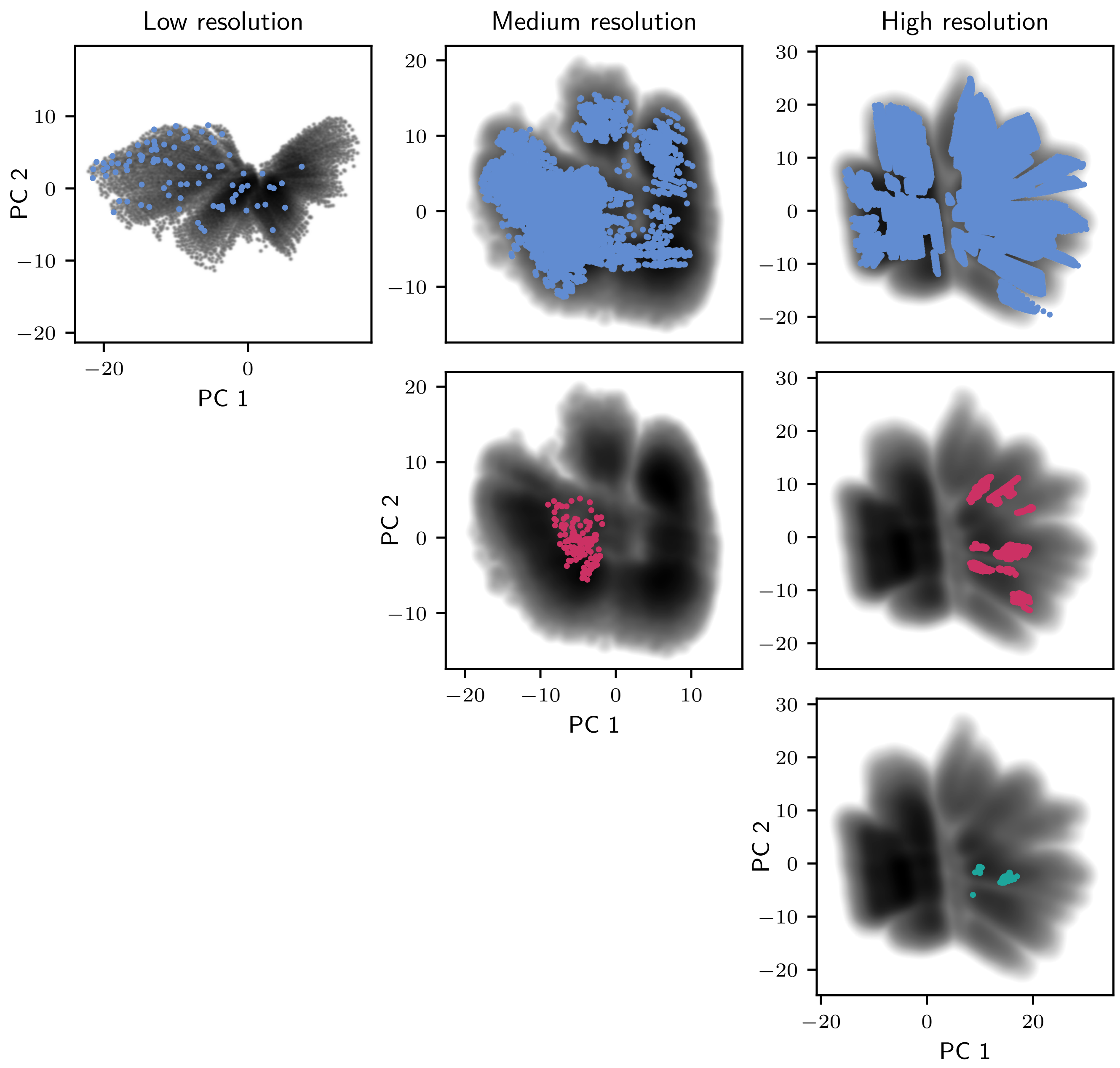

FIG. 2.10. Latent space mapping of evaluated molecules across different CS resolutions. The diagonal plots display the evaluated molecules at low, medium, and high resolution, respectively (colored points), overlaid on a kernel density estimation (black background) of the full latent representation of CS. Off-diagonal plots show the corresponding evaluated points mapped to higher resolutions. For example, the first row presents all molecules evaluated at low resolution alongside those at higher resolutions that map down to them. The latent spaces at different resolutions are learned independently and cannot be directly mapped onto each other. However, the hierarchical design of the CG model enables consistent mapping of individual molecules and thus their corresponding latent space points between resolutions. All plots represent 2D PCA projections of the five-dimensional latent space. The diagrams illustrate that broad coverage at lower resolutions propagates to broad coverage at higher resolutions. The rightmost column of the figure illustrates the funnel-like behavior of the optimization process, where broad coverage at lower resolutions gradually focuses towards promising regions in higher-resolution spaces.

[1] P. C. T. Souza, R. Alessandri, J. Barnoud, S. Thallmair, I. Faustino, F. Grünewald, I. Patmanidis, H. Abdizadeh, B. M. H. Bruininks, T. A. Wassenaar, P. C. Kroon, J. Melcr, V. Nieto, V. Corradi, H. M. Khan, J. Domański, M. Javanainen, H. Martinez-Seara, N. Reuter, R. B. Best, I. Vattulainen, L. Monticelli, X. Periole, D. P. Tieleman, A. H. de Vries, and S. J. Marrink, Nat. Methods **18**, 382–388 (2021).
[2] Non-bonded interactions - GROMACS 2025.1 documentation, `https://manual.gromacs.org/current/reference-manual/functions/nonbonded-interactions.html#equation-eqnsigeps`, [Accessed 03-05-2025].
[3] B. Mohr, K. Shmilovich, I. S. Kleinwächter, D. Schneider, A. L. Ferguson, and T. Bereau, Chem. Sci. **13**, 4498–4511 (2022).
[4] itertools — Functions creating iterators for efficient looping, `https://docs.python.org/3/library/itertools.html`, [Accessed 04-05-2025].
[5] W. L. Hamilton, R. Ying, and J. Leskovec, Inductive representation learning on large graphs (2017), arXiv:1706.02216.
[6] K. Cho, B. van Merrienboer, C. Gulcehre, D. Bahdanau, F. Bougares, H. Schwenk, and Y. Bengio, Learning phrase representations using rnn encoder-decoder for statistical machine translation (2014), arXiv:1406.1078.
[7] J. Gilmer, S. S. Schoenholz, P. F. Riley, O. Vinyals, and G. E. Dahl, (2017), arXiv:1704.01212.
[8] O. Vinyals, S. Bengio, and M. Kudlur, Order matters: Sequence to sequence for sets (2015), arXiv:1511.06391.
[9] P. Ghosh, M. S. M. Sajjadi, A. Vergari, M. Black, and B. Scholkopf, in *International Conference on Learning Representations* (2020).
[10] A. Paszke, S. Gross, F. Massa, A. Lerer, J. Bradbury, G. Chanan, T. Killeen, Z. Lin, N. Gimelshein, L. Antiga, A. Desmaison, A. Kopf, E. Yang, Z. DeVito, M. Raison, A. Tejani, S. Chilamkurthy, B. Steiner, L. Fang, J. Bai, and S. Chintala, in *Advances in Neural Information Processing Systems*, Vol. 32 (Vancouver, Canada, 2019).
[11] M. Fey and J. E. Lenssen, in *ICLR Workshop on Representation Learning on Graphs and Manifolds* (New Orleans, USA, 2019).
[12] B. Xu, N. Wang, T. Chen, and M. Li, Empirical evaluation of rectified activations in convolutional network (2015), arXiv:1505.00853.
[13] D. P. Kingma and J. Ba, Adam: A method for stochastic optimization (2014), arXiv:1412.6980.
[14] A. Centi, A. Dutta, S. H. Parekh, and T. Bereau, Biophys. J. **118**, 1321–1332 (2020).
[15] M. J. Abraham, T. Murtola, R. Schulz, S. Páll, J. C. Smith, B. Hess, and E. Lindahl, SoftwareX **1–2**, 19–25 (2015).
[16] S. Páll, A. Zhmurov, P. Bauer, M. Abraham, M. Lundborg, A. Gray, B. Hess, and E. Lindahl, J. Chem. Phys. **153**, 10.1063/5.0018516 (2020).
[17] M. Bernetti and G. Bussi, J. Chem. Phys. **153**, 10.1063/5.0020514 (2020).
[18] H. Kim, B. Fábián, and G. Hummer, J. Chem. Theory Comput. **19**, 8919–8929 (2023).
[19] G. Fiorin, M. L. Klein, and J. Hénin, Mol. Phys. **111**, 3345–3362 (2013).
[20] J. Barnoud, G. Rossi, S. J. Marrink, and L. Monticelli, PLoS Comput. Biol. **10**, e1003873 (2014).
[21] J. R. Gardner, G. Pleiss, D. Bindel, K. Q. Weinberger, and A. G. Wilson, in *Advances in Neural Information Processing Systems* (Montréal, Canada, 2018).
